# *Proteiniphilum* and *Methanothrix harundinacea* became dominant acetate utilizers in a methanogenic reactor operated under strong ammonia stress

**DOI:** 10.3389/fmicb.2022.1098814

**Published:** 2023-01-06

**Authors:** Gao Feng, Yan Zeng, Hui-Zhong Wang, Ya-Ting Chen, Yue-Qin Tang

**Affiliations:** ^1^College of Architecture and Environment, Sichuan University, Chengdu, Sichuan, China; ^2^Institute of New Energy and Low-Carbon Technology, Sichuan University, Chengdu, Sichuan, China; ^3^Institute for Disaster Management and Reconstruction, Sichuan University, Chengdu, Sichuan, China; ^4^Sichuan Environmental Protection Key Laboratory of Organic Wastes Valorization, Chengdu, Sichuan, China; ^5^Engineering Research Center of Alternative Energy Materials & Devices, Ministry of Education, Chengdu, Sichuan, China

**Keywords:** anaerobic digestion, ammonia inhibition, microbial community, syntrophic acetate oxidation, methanogenic pathways, energy conservation, anti-oxidative stress, meta-omics

## Abstract

Microorganisms in anaerobic digestion (AD) are easily affected by ammonia, especially acetoclastic methanogens. Thus, in ammonia-suppressed AD systems, acetate degradation is reported to be carried out mainly by the cooperation of syntrophic acetate oxidizers and hydrogenotrophic methanogens. Previous studies have revealed ammonia inhibition on microbial flora by AD performance, but the effect mechanism of ammonia on microbial metabolism remains poorly understood. In this study, we constructed a mesophilic chemostat fed with acetate as the sole carbon source, gradually increased the total ammonia nitrogen (TAN) concentration from 1 g L^−1^ to 6 g L^−1^, and employed the 16S rRNA gene, metagenomics, and metatranscriptomics analysis to characterize the microbial community structure and metabolic behavior. The results showed that even at the TAN of 6 g L^−1^ (pH 7.5), the methanogenesis kept normal, the biogas production was approximately 92% of that at TAN of 1 g L^−1^ and the acetate degradation ratio reached 99%, suggesting the strong TAN tolerance of the microbial community enriched. 16S rRNA gene analysis suggested that the microbial community structure changed along with the TAN concentration. *Methanothrix* predominated in methanogens all the time, in which the dominant species was gradually replaced from *M. soehngenii* to *M. harundinacea* with the increased TAN. Dominant bacterial species also changed and *Proteiniphilum* showed a significant positive correlation with increased TAN. Meta-omics analysis showed that the absolute dominant microorganisms at TAN of 6 g L^−1^ were *M. harundinacea* and *Proteiniphilum*, both of which highly expressed genes for anti-oxidative stress. *M. harundinacea* and the second dominant methanogen *Methanosarcina* highly expressed both acetate cleavage and CO_2_ reduction pathways, suggesting the possibility that these two pathways contributed to methanogenesis together. *Proteiniphilum* and some other species in Firmicutes and Synergistetes were likely acetate oxidizers in the community as they highly expressed genes for syntrophic acetate oxidization, H_2_ generation, and electron transfer. These results suggested that *Proteiniphilum* as well as *M. harundinacea* have strong ammonia tolerance and played critical roles in acetate degradation under ammonia-suppressed conditions. The achievements of the study would contribute to the regulation and management of the AD process.

## 1. Introduction

Anaerobic digestion (AD) is a core technology for organic waste treatment that generates methane as a renewable clean biofuel. However, AD is easily affected by various inhibitors, of which ammonia (NH_3_) and ammonium (NH_4_^+^) are the most prominent inhibitors (Hansen et al., 1998; Yenigün and Demirel, 2013). Although appropriate ammonium is beneficial for AD stability, undesirably high concentrations may be reached during the degradation of organic waste, especially those with high protein and urea content. It is generally agreed that the main factor leading to AD inhibition is ammonia, which causes proton imbalance and potassium loss and affects microbial metabolism (Gallert et al., 1998). McCarty and McKinney (1961) found that 0.15 g L^−1^ NH_3_ was completely inhibitory to AD. Another study showed that the total ammonia nitrogen (TAN) of around 1.7–1.8 g L^−1^ caused AD failure (Melbinger et al., 1971). Therefore, studying the effects of ammonia or TAN on AD microorganisms is crucial for developing countermeasures to reduce inhibition and maintain stable AD operation.

Volatile fatty acids (VFAs) are the most important intermediates in the AD process, especially acetate, which is the source of 70–80% of methane (Mackie and Bryant, 1981). As is well known, acetate can be converted to methane through two routes: acetate cleavage (Eq. 1) and acetate oxidation (Eqs 2, 3).


(1) $$CH_{3}COO^{-}+H_{2}O\to CH_{4}+HCO_{3}{}^{-}\Delta G^{0\prime }=-31.0\;kJ/mol$$

(2) $${\mathrm{CH}}_{3}{\mathrm{COO}}^{-}+4H_{2}O\to 2{\mathrm{HCO}}_{3}{}^{-}+4H_{2}+H^{+}\Delta G^{0\prime }=+104.6\;\mathrm{kJ}/\mathrm{mol}$$

(3) $$4H_{2}+{\mathrm{HCO}}_{3}{}^{-}+H^{+}\to {\mathrm{CH}}_{4}+3H_{2}O\Delta G^{0\prime }=-135.6\;\mathrm{kJ}/\mathrm{mol}$$

Because of the thermodynamically unfavorable energetics, acetate oxidation strongly depends on the removal of H_2_ by cooperating with partner hydrogenotrophic methanogens (Hattori, 2008). Such syntrophic cooperation is known to provide limited energy for the growth of two microbes, resulting in a low growth rate. However, previous studies have found that ammonia inhibits methanogens, especially acetate-consuming methanogens (Westerholm et al., 2012). Thus, under ammonia-inhibition conditions, syntrophic acetate oxidation is reported to become the main methanogenesis pathway (Schnürer and Nordberg, 2008). Jiang et al. (2018) used ^14^C radiolabeled acetate to investigate the link between TAN and the methanogenesis pathways. The results showed that the proportions of hydrogenotrophic methanogenesis in the high (11.1 g/kg) and low (0.2 g/kg) TAN concentration conditions were 68–75% and 9–23%, respectively, indicating the strong ammonia tolerance of syntrophic acetate oxidation methanogenesis pathway.

Therefore, studying the acetate degradation and methanogenesis pathways under ammonia stress is crucial for regulating AD and attenuating inhibition. Previous studies have revealed ammonia inhibition on microbial flora by AD performance; however, the effect mechanism of ammonia on microbial metabolism is not yet clear. Meanwhile, most previous studies were performed using batch cultures, which differed from the generally continuous state of the actual AD process. Therefore, in the present study, we constructed a mesophilic chemostat fed with acetate as the sole carbon source and gradually increased the TAN concentration for acclimation. We employed the 16S rRNA gene, metagenomics, and metatranscriptomics analysis to characterize the microbial community structure and metabolic behavior of syntrophs and methanogens supporting anaerobic acetate degradation. This study provides a basis for developing technology to improve ammonia tolerance and the operational stability of AD.

## 2. Materials and methods

### 2.1. Chemostat operation and performance

The acetate-degrading anaerobic chemostat was constructed using a continuous stirred tank reactor (CSTR) with a working volume of 1.8 l that was mixed using a magnetic stirrer at 300–400 rpm (Supplementary Figure S1). The reactor was immersed in a thermostat-controlled water bath to maintain a temperature of 37°C. The seed sludge from a swine manure treatment plant (Sichuan Province, China) was used to inoculate the reactor. Artificial wastewater containing acetate as the sole carbon source was continuously supplied to the CSTR by a peristaltic pump under an atmosphere of N_2_, and the effluent flowed out from a U-type tube (Supplementary Figure S1). The wastewater [total organic carbon (TOC) concentration of 8.0 g L^−1^] contained the following components per liter of distilled water: sodium acetate, 5.46 g; acetic acid, 16.0 g; KH_2_PO_4_, 0.3 g; KHCO_3_, 4.0 g; NH_4_Cl, 1.0 g; NaCl, 0.6 g; MgCl_2_·6H_2_O, 0.82 g; CaCl_2_·2H_2_O, 0.08 g; and cysteine-HCl·H_2_O, 0.1 g, supplemented with 10 ml trace element solution of DSMZ medium 318 (Deutsche Sammlung von Mikroorganismen und Zellkulturen, Germany) comprising 1.19 mg L^−1^ of Ni^2+^ and 0.34 mg L^−1^ of Co^2+^, and 10 ml vitamin solution of DSMZ medium 318 without vitamin B_12_.

The reactor was operated at a dilution rate of 0.05 d^−1^. After the system reached steady operation, additional NH_4_Cl was added to gradually increase TAN concentration from 1 g L^−1^ to 6 g L^−1^. Biogas production, gas composition, pH, concentrations of volatile suspended solids (VSS), suspended solids (SS), TOC, as well as VFAs were measured periodically using the protocols reported previously (Chen et al., 2020b). Microbial morphology at each stage was observed using a laser-scanning confocal fluorescence microscope (Olympus FV1000, Japan). During the steady operation period at each TAN concentration (N0 ~ N6), biomass was collected from the broth and used for DNA and RNA extraction.

### 2.2. DNA extraction, 16S rRNA gene PCR, Illumina sequencing, and data processing

To investigate the microbial community, 40 ml broth from the chemostat (days 103, 138, 187, 222, 250, 301, and 390) was collected in sterile DNase-free centrifuge tubes, centrifuged at 10,000 rpm at 4°C for 10 min, and rinsed thrice with sterile phosphate buffer saline (PBS) (10 mM, pH 7.5). Total DNA was extracted *via* the cyltrimethyl ammonium bromide (CTAB) method (Griffiths et al., 2000). DNA extracts were subjected to 16S rRNA gene amplicon sequencing. The V4-V5 hypervariable regions of both bacterial and archaeal 16S rRNA genes were amplified using universal primers 515F (5’-GTGCCAGCMGCCGCGGTAA-3′) and 909R (5’-CCCCGYCAATTCMTTTRAGT-3′). PCR product purification, Illumina sequencing, and data processing were conducted as described previously (Zheng et al., 2019). The raw reads of the 16S rRNA gene sequencing were deposited into the NCBI Sequence Read Archive (SRA) database with the accession number PRJNA524473.

### 2.3. Metagenomics and metatranscriptomics sequencing and bioinformatics analyses

To analyze the metabolic characteristics of the community under high ammonia stress (TAN concentration of 6 g L^−1^), sludge samples for metagenomics and metatranscriptomics sequencing were collected on days 390 and 393. Total DNA and RNA were extracted *via* the CTAB method (Griffiths et al., 2000). The metagenome DNA was sequenced on an Illumina HiSeq 3,000 platform (Illumina). The obtained raw data were trimmed *via* Trimmomatic v0.36 (Bolger et al., 2014) with a quality cutoff of 30, a sliding window of 6 bp, and a minimum length cutoff of 100 bp. The clean reads from two metagenomes corresponding were co-assembled *via* SPAdes v.3.5.0 (Bankevich et al., 2012), binned using MetaBAT (Kang et al., 2015), checked for completeness and contamination using CheckM (Parks et al., 2015), and calculated relative abundance using BBMap (Bushnell, 2014). Phylogenomic trees were built with PhyloPhlAn v0.99 (“-u” option) (Segata et al., 2013). Genes were then annotated using Prokka (Seemann, 2014) and manual curation was performed as described previously (Chen et al., 2020a).

For metatranscriptomics sequencing, total RNA was treated with DNase to remove the residual DNA using an RNase-free DNase set (Qiagen, Hilden, Germany). Ribosomal RNA (rRNA) was removed from the DNase-treated RNA *via* the Ribo-Zero rRNA Removal Kits (Illumina, San Diego, CA, United States). RNAseq libraries were created using the TruSeq RNA sample prep kit (Illumina, San Diego, CA, United States) with the standard protocol. The sample libraries were sequenced on an Illumina HiSeq 3,000 sequencer. The metatranscriptomics sequences were trimmed as the DNA-trimming step described above and mapped to metagenomic bins using the Bowtie2 aligner (Langmead and Salzberg, 2012). The expression levels of given genes from each bin were calculated separately as reads per kilobase transcript per million reads (RPKM) mapped to the bin averaged from the duplicate samples. In the heatmap illustration, the gene expression levels were further normalized to the median gene expression levels of each bin (RPKM-NM) averaged from the duplicate samples (Nobu et al., 2017). The raw reads of the metagenomics and metatranscriptomics sequencing are accessible at http://bigd.big.ac.cn/gsa, accession number: CRA008529.

## 3. Results

### 3.1. Chemostat operation and performance

**Figure 1:**
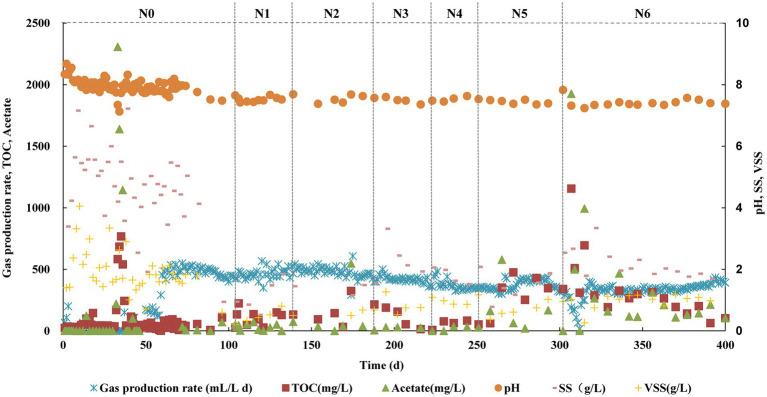
Performance of the anaerobic chemostat fed with acetate as the sole carbon source under different TAN concentrations.

The reactor operated continuously for more than 400 days and the methanogenesis kept stable during each stage with different TAN concentrations (N0 ~ N6 stages). The pH was stable at about 7.5 at all stages. From N0 to N2 stages, the biogas production did not decrease, and the acetate fed was completely degraded. From N3, the biogas production decreased slightly, but at the N6 stage, it was about 92% of that at N0 ~ N2 (Figure 1). In biogas, CH_4_ accounted for 53–59%, CO_2_ accounted for 41–47%, and the partial pressure of H_2_ was less than 1 Pa. Acetate built up in the initial period of N5 and N6, but with the extension of the running time, it was degraded. The acetate degradation ratio was high of 99% at the N6 stage. The SS and VSS concentrations increased slightly with increased TAN which was around 1.69 and 0.98 g L^−1^, respectively, at stage N6 (Figure 1). These results suggested that the methanogenesis was not repressed obviously even at a TAN of 6 g L^−1^ (free ammonia nitrogen concentration of 0.23 g L^−1^).

We performed fluorescence observation of the microbial community at each stage. F_420_, the unique key coenzyme in the hydrogenotrophic methanogenesis pathway, can make cells fluoresce blue-green under UV light at 420 mm wavelength (Braks et al., 1994). During the N0 to N4 stages, the microbial morphology was mainly tubular, and almost no fluorescence cell was detected (Supplementary Figure S2). When the TAN concentration reached 5 g L^−1^, spherical cells were clearly observed, and a strong fluorescence was detected (Supplementary Figure S2). This result indicated that with the increase of TAN concentration, cells able to produce methane through the hydrogenotrophic pathway probably increased, which also suggested that acetate oxidation probably occurred in the reactor.

### 3.2. Microbial diversity and community composition of acetate-fed chemostat under ammonia inhibition

**Figure 2:**
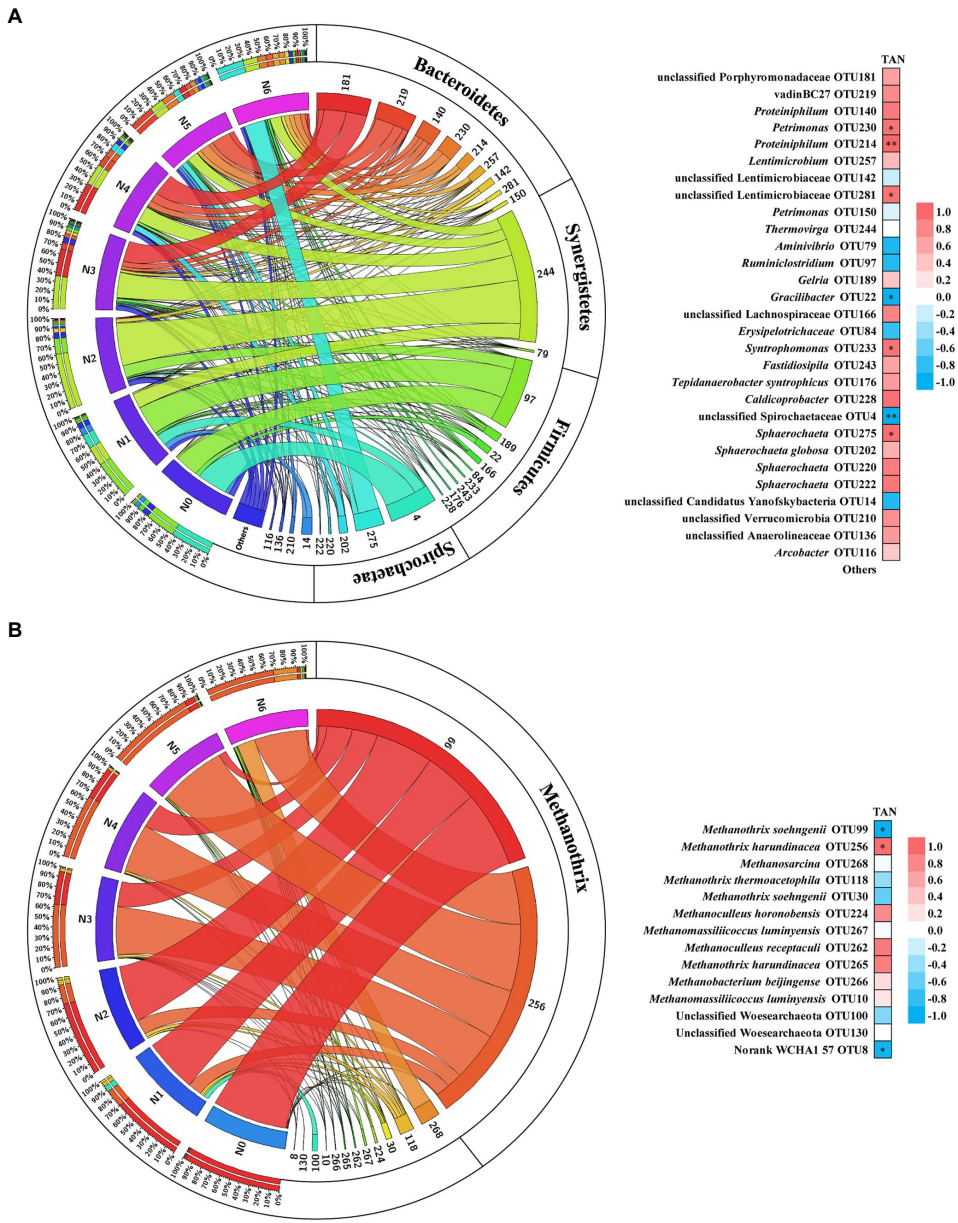
Relative abundance in different stages and the Spearman’s correlation with TAN concentration of bacterial **(A)** and archaeal **(B)** OTUs. Ribbon width represents the relative abundance. Red represents a positive correlation; blue represents a negative correlation; significant correlations (value of *p* ≤ 0.05) are labeled by *; extremely significant correlations (value of *p* ≤ 0.01) are labeled by **.

The microbial community analysis was carried out using the sludge samples on the last day of each operation stage (N0 ~ N6). Based on 16S ribosomal RNA gene analysis, the structure of the microbial community varied greatly with increased TAN concentration (Supplementary Figure S3). Compared with bacteria, the archaeal populations were significantly inhibited, and the relative abundance decreased to 3.54% during the stage of N3 and N4 (Supplementary Figure S4). As the TAN concentration continued to be increased, the relative abundance of archaea gradually recovered to 18.35%. To further reveal the impact of TAN on microbial communities, the dominant bacterial and archaeal (>1% relative abundance at any stage) communities at the operational taxonomic units (OTU) level were analyzed and Spearman’s correlation coefficients were calculated to illustrate the correlation between microbial community structure and TAN concentration (Figure 2). The dominant bacterial phyla included Bacteroidetes, Synergistetes, Firmicutes, and Spirochaetae (Figure 2A; Supplementary Figure S6A). OTUs in Bacteroidetes prevailed in high TAN conditions, in which the relative abundance of *Petrimonas* (OTU230) and unclassified Lentimicrobiaceae (OTU281) were significantly positively correlated with TAN concentration and *Proteiniphilum* (OTU214) was extremely significantly correlated, suggesting the high TAN tolerance of these OTUs. In the Synergistetes phylum, the *Thermovirga* (OTU244) predominated in the N2 stage (61%), but the relative abundance continued to decrease in the later stages. The most abundant OTU in Firmicutes was *Ruminiclostridium* (OTU97), which was negatively correlated with TAN concentration, while syntrophic butyrate (*Syntrophomonas* [OTU233]) and acetate (*Tepidanaerobacter syntrophicus* [OTU176])-oxidizing bacteria were positively correlated with TAN. The main genera in Spirochaetae were unclassified *Spirochaetaceae* (OTU4) and *Sphaerochaeta* (OTU275, 202, 220, and 222), their responses to TAN stress are diametrically opposite. Unclassified Spirochaetaceae (OTU4) was strongly inhibited by TAN, whereas *Sphaerochaeta* was not.

Regarding the archaeal community, the OTUs were determined at the species level by phylogenetic analysis (Supplementary Figure S5). Among them, *Methanothrix* was the most abundant methanogen across all the stages, the genus mainly included *M. soehngenii* (OTU99) and *M. harundinacea* (OTU256), which showed totally opposite trends (Figure 2B). With the increase of TAN concentration, the dominant methanogen in the chemostat was gradually replaced from *M. soehngenii* (OTU99) to *M. harundinacea* (OTU256), suggesting that *M. harundinacea* was more tolerant to TAN suppression. Moreover, the diversity of methanogens increased significantly with the increase in TAN concentration. *Methanosarcina* (OTU 268) was only detected in the N6 stage, and its relative abundance accounted for 23.5% of total archaeal species (Figure 2B; Supplementary Figure S6B).

**Figure 3:**
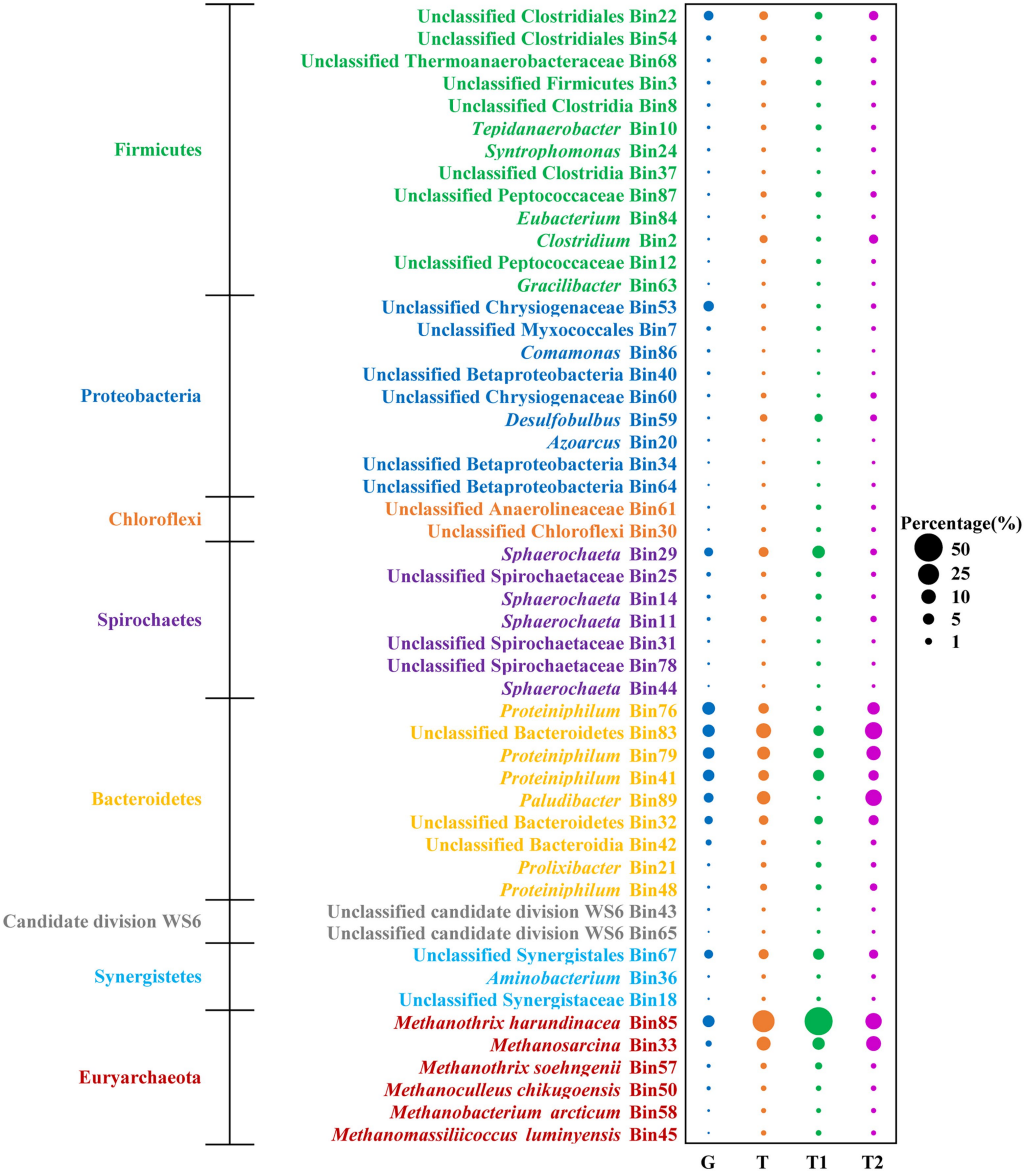
Metagenomic abundance (G) and percentage of metatranscriptomic reads (T) of bacterial and archaeal bins. The corresponding abundance of genomic bins in the N6 stage are estimated from their metagenomic coverage calculated as the percentage of metagenomics (MG) reads mapped to each genome relative to the total reads mapped to all constructed bacterial and archaeal genomes. The estimated activity of these genomes is shown as the percentage of metatranscriptomic (MT) reads mapped to each genome relative to the total reads mapped to all constructed bacterial and archaeal genomes. G, total MG reads; T, total MT reads; T1, MT reads of sampling time point 1; T2, MT reads of sampling time point 2.

To profile the metabolic capability of TAN-tolerant bacteria and methanogens, metagenomics analysis was performed on microbial community samples taken from the chemostat at the N6 stage on two different dates. Overall, 34 Gbp metagenomic clean sequences were obtained. Illumina paired-end reads from the two duplicate samples were co-assembled, and binning the assembled contigs of metagenomes yielded 89 bins. Among these, 45 bacterial and 6 archaeal bins which have at least 72% genome completeness and < 6.5% contamination as estimated by CheckM (Parks et al., 2015) were analyzed (Supplementary Figures S7, S8; Supplementary Table S1). To further reveal the metabolic behavior of the microbial populations, 56.6 million metatranscriptomic reads (4.5 Gbp) were produced and mapped to the metagenomic bins. The percentages of metatranscriptomic reads mapped to dominant genomes were, respectively, analyzed at two sampling time points, and the two transcriptomics analyses displayed similar results (Figure 3). Based on mapping meta-omic reads to the obtained bins, the bacterial populations retrieved accounted for 86 and 60% of the metagenomic and metatranscriptomic reads, respectively (Supplementary Figure S9). Among all bacterial phyla, *Bacteroidetes* represented major fractions of the 16S rRNA (40.85%), metagenomes (49.59%), and metatranscriptomes (39.15%) at the N6 stage (Figure 3; Supplementary Figures S6A). The major genera in Bacteroidetes were *Proteiniphilum* (Bin76, Bin79, and Bin41), unclassified Bacteroidetes (Bin83 and Bin32), and *Paludibacter* (Bin 89; (Figure 3). In addition, as observed in the 16S rRNA gene analysis, populations associated with known syntrophic fatty acid-oxidizing bacteria (*Tepidanaerobacter* Bin10 and *Syntrophomonas* Bin24) were detected in Firmicutes (Figures 2A, 3).

As for the methanogen, *M. harundinacea* (Bin85) was the most abundant, accounting for 9.10 and 28.07% of the metagenomic and metatranscriptomic reads, respectively (Figure 3). The second dominant methanogen was *Methanosarcina* (Bin33) with 1.95% metagenomic abundance and 8.98% metatranscriptomic abundance. It is worth mentioning that *M. soehngenii* (Bin57) only accounted for 0.58 and 0.65% of the metagenomic and metatranscriptomic reads, consistent with the results of 16S rRNA gene analysis (Figures 2B, 3).

### 3.3. Methanogenic pathways and energy-conserving metabolisms in methanogens

**Figure 4:**
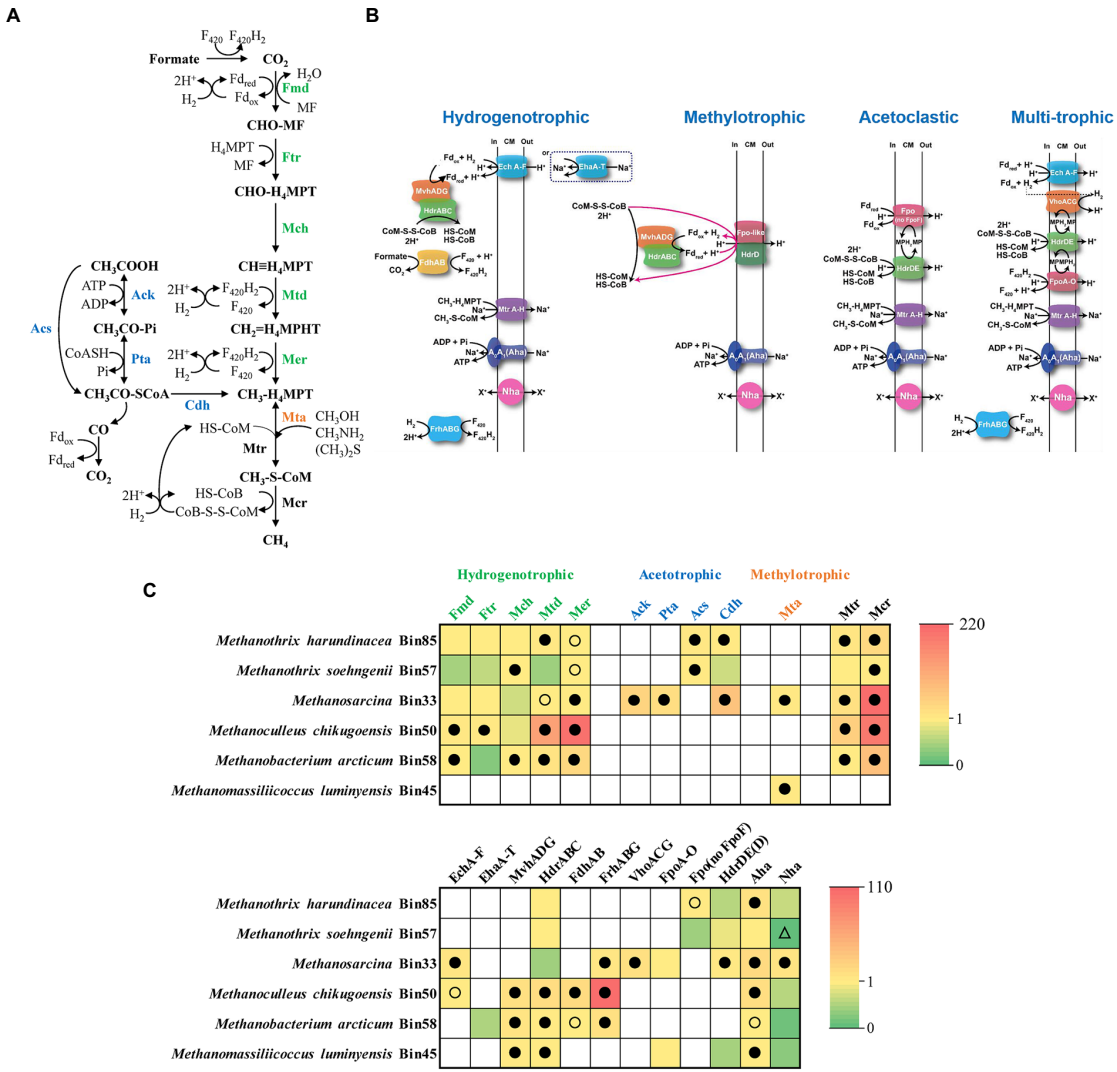
Metabolic pathways of methanogenesis **(A)**, methanogenic electron transfer **(B)**, and the gene expression level **(C)** of methanogens in the N6 stage. The gene expression levels are calculated as reads per kilobase of transcript per million reads mapped to individual bins (RPKM) normalized to the median gene expression for the corresponding bin (RPKM-NM) averaged from duplicate samples. Pathways containing genes with RPKM-NM of 0 and greater than the octile and quartile are marked (open triangle, filled and open dots, respectively). Enzyme abbreviations and their corresponding genes are elaborated in Supplementary Tables S2, S3.

The community hosted diverse methanogens, including *M. harundinacea* (Bin85), *M. soehngenii* (Bin57), *Methanosarcina* (Bin33), *Methanoculleus chikugoensis* (Bin50), *Methanobacterium arcticum* (Bin58), and *Methanomassiliicoccus luminyensis* (Bin45) (Supplementary Figure S8; Supplementary Table S1). These methanogens have distinct substrate spectrums for methane production, including the acetate cleavage pathway, CO_2_ reduction pathway, and methyl cleavage pathway (Figure 4A; Supplementary Table S2). During methanogenesis, methanogens synthesize ATP *via* a transmembrane Na^+^/H^+^ gradient formed by an electron transfer chain (Figure 4B; Supplementary Table S3). To further understand the metabolic profile of methanogens, we analyzed the methanogenic and energy metabolic pathways of target bins by comparison with known pathways in the UniProt database, then mapped the metatranscriptomic reads to bins and calculate RPKM-NM to analyze the expression level of target pathways (Figure 4C).

Specifically*, M. harundinacea* (Bin85) and *Methanosarcina* (Bin33) expressed acetate utilization pathway highly (top octile of expressed genes in the corresponding bin) and *M. soehngenii* (Bin57) expressed at a lower level (Figures 4A,C; Supplementary Table S2). The *M. harundinacea* and *Methanosarcina* correspondingly highly expressed (top quartile or octile) genes for acetoclastic methanogenesis mediated by Fpo (no FpoF) and H_2_-cycling (*via* EchA-F and VhoACG; Figures 4B,C; Supplementary Table S3; Welte and Deppenmeier, 2014).

Bins of hydrogenotrophic methanogens, such as *M. chikugoensis* (Bin50), *M. arcticum* (Bin58), and *Methanosarcina* (Bin33), encode and express genes for CO_2_-reducing methanogenesis (Figures 4A,C; Supplementary Table S2). The *Methanoculleus* highly expressed (top quartile or octile) genes for the oxidation of H_2_ (MvhADG-HdrABC and FrhABG) and formate (FdhAB; Figures 4B,D; Supplementary Table S3). Another H_2_-oxidizing population, *M. luminyensis* (Bin45), highly expressed genes for coupling of H_2_ oxidation with the reduction of methanol and dimethylamine to methane (Figure 4; Supplementary Tables S2, S3). Interestingly, *Methanothrix* is a well-known obligate acetotrophic methanogen (Hattori, 2008), but we found the expression of the CO_2_-reducing methanogenesis pathway was high in *Methanothrix*, especially in Bin85 (Figures 4A,C; Supplementary Table S2; Holmes et al., 2017). However, *Methanothrix* did not express the key genes for utilizing H_2_ (Figures 4B,C), it may directly use extracellular electrons for reducing CO_2_ to CH_4_ (Rotaru et al., 2014; Zhao et al., 2020). Thus, the acetate-fed chemostat was likely to produce methane not only *via* acetoclastic pathway but also possible *via* the CO_2_ reduction pathway under high TAN-suppressed conditions.

### 3.4. Syntrophic metabolism and energy conservation of acetate-oxidizing community

**Figure 5:**
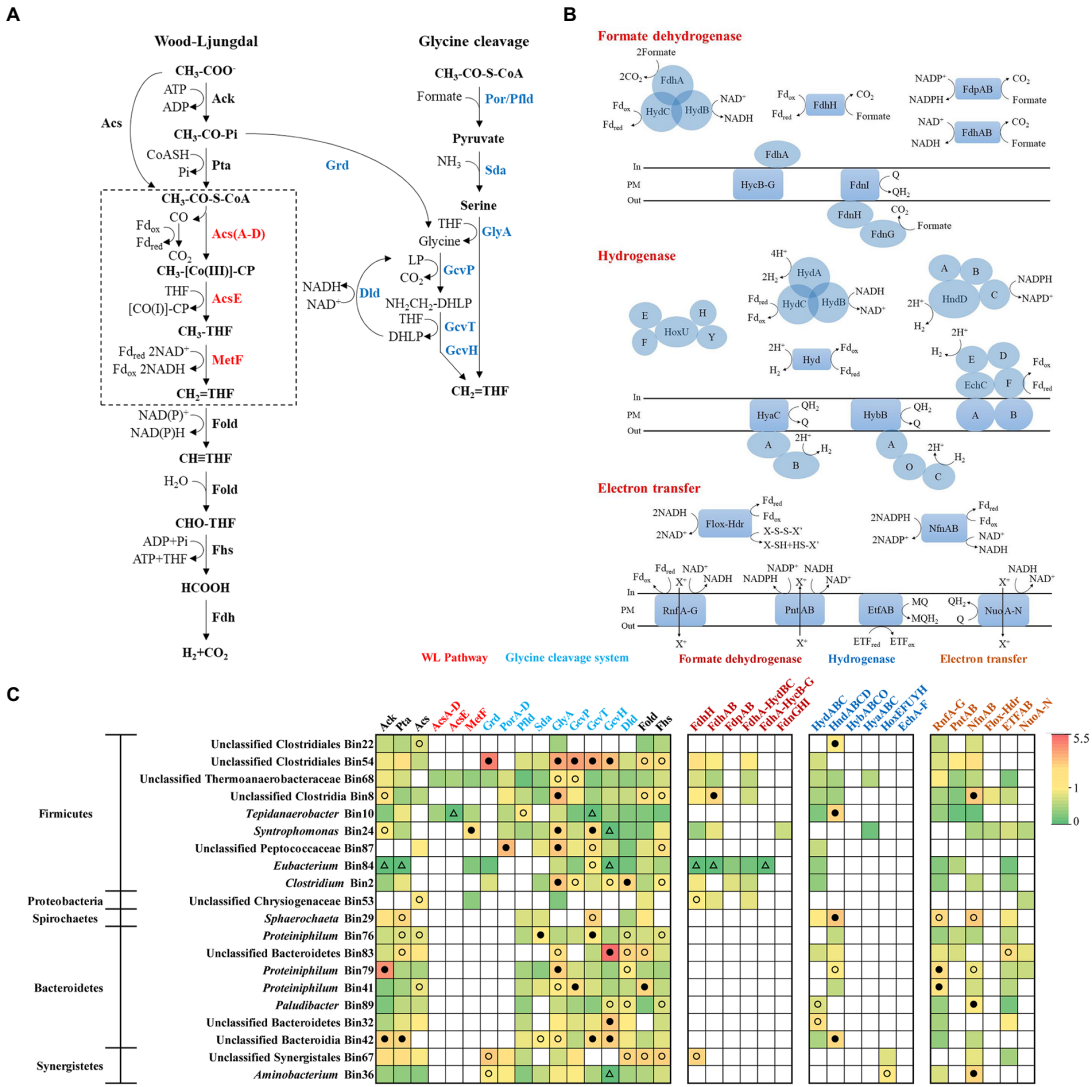
Metabolic pathways of acetate oxidation **(A)**, H_2_/formate metabolism and electron transfer **(B)**, and the gene expression level **(C)** of syntrophs which may syntrophically degrade acetate in the N6 stage. The gene expression levels are calculated as reads per kilobase of transcript per million reads mapped to individual bins (RPKM) normalized to the median gene expression for the corresponding bin (RPKM-NM) averaged from duplicate samples. Pathways containing genes with RPKM-NM of 0 and greater than the octile and quartile are marked (open triangle, filled and open dots, respectively). Enzyme abbreviations and their corresponding genes are elaborated in Supplementary Tables S4, S5.

As discussed above, in the acetate-fed and TAN-suppressed methanogenic system, besides directly decomposing acetate into methane, methanogens can also use the products (electron, H_2_, and CO_2_) of syntrophic acetate oxidizers to generate methane. There are currently two acetate oxidation pathways reported (Zhu et al., 2020), the reversed Wood–Ljungdahl pathway and the glycine cleavage pathway, which are different in the process from CH_3_-CO-S-CoA to CH_2_ = THF (Figure 5A). To systematically analyze the potential acetate-oxidizing bacteria in the system, 45 bacterial bins were annotated and then compared with the known acetate-oxidizing pathways in the UniProt database (Supplementary Figure S10; Supplementary Table S4). We screened out 20 bacterial bins with complete acetate oxidation pathway or metagenomic abundance greater than 1%, then mapped the metatranscriptomic reads to bins and calculated RPKM-NM to analyze the expression levels of genes related to acetate oxidation, H_2_/formate metabolism, and electron transport (Figure 5).

Within the acetate-degrading community, unclassified Thermoanaerobacteraceae (Bin68) encode all genes for both reversed Wood–Ljungdahl and glycine cleavage pathways with high expression of GlyA and GcvP (top quartile of expressed genes in the corresponding bin; Figures 5A,C; Rui et al., 2011). A population (Bin10) related to a known acetate-degrading genus, *Tepidanaerobacter*, expressed conversion of acetate to formate through the reversed Wood–Ljungdahl pathway (Figures 5A,C; Westerholm et al., 2011). Another population (Bin54) belongs to Clostridiales expressed genes for acetate degradation (Grd, GlyA, GcvP, GcvT, and GcvH) at high levels (top octile; Figure 5C; Westerholm et al., 2018). Notably, *Proteiniphilum* (Bin76, Bin79, and Bin41) had the highest abundance in both metagenomic and metatranscriptomic reads (Figure 3) and highly expressed genes of glycine cleavage pathway (top quartile or octile; Figures 5A,C); thus, these populations are likely novel acetate degraders.

Multiple genes/gene clusters encoding formate dehydrogenase and hydrogenase are detected in the aforementioned syntrophs for formate and H_2_ generation. These enzymes are necessary for the reoxidation of reducing equivalents (i.e., menaquinone, NADH, NADPH, and reduced ferredoxin [Fd]) generated during acetate oxidation (Figures 5B; Supplementary Table S5). For formate metabolism, all syntrophic metabolizers in Firmicutes expressed Fd-dependent formate dehydrogenases (FdhH), NADH-dependent formate dehydrogenases (FdhAB), and putative electron-confurcating formate dehydrogenases (FdhA-HydBC) except for *Tepidanaerobacter* (Bin10) and unclassified *Peptococcaceae* (Bin87; Figures 5B,C; Supplementary Figure S11). Among them, unclassified Clostridia Bin8 highly expressed FdhAB (top octile; Figure 5C). For H_2_ generation, all syntrophic metabolizers in Firmicutes (except for *Syntrophomonas*) expressed cytoplasmic [FeFe]-type electron-confurcating hydrogenases (HydABC) (Figures 5B,C; Supplementary Figure S11). These hydrogenases catalyze the thermodynamically favorable production of H_2_ from Fd_red_ to drive the unfavorable production of H_2_ from NADH (Buckel and Thauer, 2013). We detected high expression of [FeFe]-type NADP^+^-dependent hydrogenases (HndABCD) in unclassified *Clostridiales* (Bin22), *Tepidanaerobacter* (Bin10), *Sphaerochaeta* (Bin29), *Proteiniphilum* (Bin79), and unclassified Bacteroidia (Bin42) (top octile or quartile; Figure 5C; de Luca et al., 1998).

To support the thermodynamically challenging H_2_ and formate generation, the syntrophs also expressed energy-conserving electron transfer enzymes. The *Proteiniphilum* populations (Bin79 and Bin41) highly expressed (top octile) the membrane-bound *Rhodobacter* nitrogen fixation complex (RnfA-G), which can either extrude cation and transfer an electron from Fd_red_ to NAD^+^ to gain energy or transport cation inward and transfer electron from NADH to Fd_ox_ to drive electron-confurcating hydrogen or formate generation (Figures 5B,C; Biegel et al., 2011). In addition, *Proteiniphilum* (Bin79) also highly expressed (top quartile) the NADP^+^-Fd_red_ oxidoreductase (NfnAB), which transfers electrons from NADPH to NAD^+^ and Fd_ox_ *via* electron disproportionation (Figures 5B,C; Wang et al., 2010).

### 3.5. Anti-oxidative stress in syntrophs and methanogens

**Figure 6:**
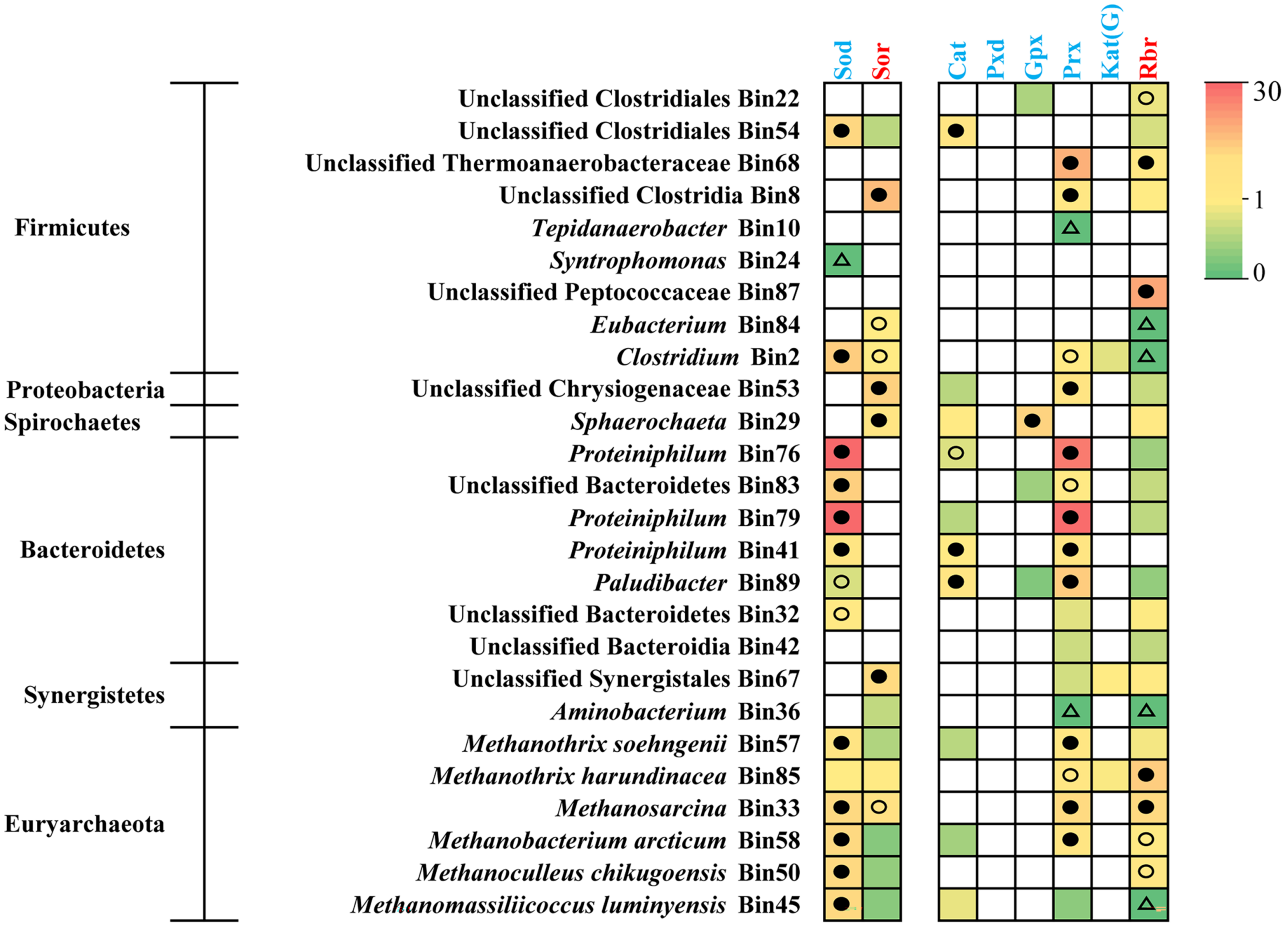
Gene expression level for anti-oxidative stress in syntrophs and methanogens. The gene expression levels are calculated as reads per kilobase of transcript per million reads mapped to individual bins (RPKM) normalized to the median gene expression for the corresponding bin (RPKM-NM) averaged from duplicate samples. Pathways containing genes with RPKM-NM of 0 and greater than the octile and quartile are marked (open triangle, filled and open dots, respectively). Enzyme abbreviations and their corresponding genes are elaborated in Supplementary Table S6.

Since the ROS-detoxification mechanism supported the adaptation of *Proteiniphilum* species (Wu et al., 2021) and ammonia caused oxidative stress in methanogens (Kato et al., 2014; Li et al., 2022), we also examined the occurrence of antioxidant genes in target bins (Figure 6; Supplementary Table S6). All the populations of *Proteiniphilum* highly expressed genes (top octile of expressed genes in the corresponding bin) encoding superoxide dismutase (Sod) and peroxiredoxins (Prx), respectively, which belong to energy-free ROS scavenger (Supplementary Table S6; Martins et al., 2019). For methanogens, in addition to detecting highly expressed Sod and Prx, we also identified the expression of energy-dependent ROS scavengers (superoxide reductase, Sor; rubrerythrin, Rbr) (Figure 6; Supplementary Table S6), which require additional energy sources with F_420_H_2_ as the ultimate electron donor (Martins et al., 2019). *Methanothrix harundinacea* (Bin85) and *Methanosarcina* (Bin33) which represented major fractions of the metagenomes and metatranscriptomes in methanogens highly expressed Sor and Rbr (top octile; Figures 3, 6). Thus, the predominant bacterial genus *Proteiniphilum* and methanogens in the TAN-suppressed system possessed mechanisms of anti-oxidative stress thereby providing a selective advantage under suppressive conditions.

## 4. Discussion

It was generally reported that TAN concentrations of around 1.7–1.8 g L^−1^ were completely inhibitory to AD under unacclimated inoculation conditions, and the TAN level could increase up to 5 g L^−1^ with acclimation (Albertson, 1961; Melbinger et al., 1971). In this study, the continuous anaerobic digestion reactor fed with acetate as the sole carbon source was operated stably at a high TAN concentration (6 g L^−1^) after a long-term continuous acclimation with progressively increasing TAN. During acclimation, both bacterial and archaeal communities underwent significant species turnover (Figure 2; Supplementary Figure S3). Thus, we evaluated why under such strong TAN inhibition, *Methanothrix harundinacea* was able to dominate methanogens, and the only dominant bacteria were *Proteiniphilum* species.

As for methanogens, the only dominant genus was *Methanothrix* during the acclimation, although multitrophic *Methanosarcina* appeared at the N6 stage, *Methanothrix* still dominated. This result suggests that acetate cleavage was probably the main way of methanogenesis under high TAN stress, which to some extent contradicts the previous studies that the methanogenic pathway was transformed from acetotrophic to hydrogenotrophic under ammonia stress (Schnürer and Nordberg, 2008; Yenigün and Demirel, 2013; Gao et al., 2015; Jiang et al., 2018). In addition, based on the fluorescence of F_420_ observed in methanogens and the increasing relative abundance of bacteria (Supplementary Figures S2, S4), it is reasonable to speculate that a part of bacteria carried out acetate oxidative metabolism in the high TAN stages. To clarify this contradiction, further validation of the methanogenesis pathway with meta-omics was conducted.

*Methanothrix harundinacea* (Bin85) and *Methanosarcina* (Bin33) predominated in methanogens at the N6 stage (Figure 3). Through meta-omics analysis, we found that *M. harundinacea* highly expressed both CO_2_ reduction and acetate cleavage pathways, and the expression of acetate cleavage pathway was higher (Figure 4C), consistent with existing research results (Holmes et al., 2017). However, we did not detect the key genes responsible for utilizing H_2_ in *M. harundinacea* (Figure 4C), suggesting the possibility that *M. harundinacea* uses extracellular electrons directly to reduce CO_2_ (Rotaru et al., 2014; Zhao et al., 2020). It has been found that *Methanothrix* can reduce CO_2_ to methane by directly accepting electrons from *Geobacter* in an AD reactor supplied with granular activated carbon (Yang et al., 2019). It is possible that *M. harundinacea* is similar to multitrophic *Methanosarcina*, which may gain more energy through multiple pathways to combat ammonia stress (Hao et al., 2015). Though *M. soehngenii* is reported also be able to receive extracellular electrons for reducing CO_2_ to CH_4_ (Yang et al., 2019), possibly due to its much lower ammonia resistance (much lower expression of Sor and Rbr; Figure 6), it lost competitiveness compared with *M. harundinacea*. It has been reported that *M. soehngenii* can be completely inhibited at a TAN concentration of 560 mg L^−1^ in pure culture (Sprott and Patel, 1986). The reason why previous studies reported that the methanogenesis pathway was transformed from acetotrophic to hydrogenotrophic under ammonia stress is probably that there was no strong ammonia-tolerant *M. harundinacea* in their systems or the lack of acclimatization to ammonia (Zhang et al., 2014; Jiang et al., 2018).

Furthermore, the key genes for both energy-free and energy-dependent anti-oxidative stress mechanisms were detected in methanogens, which may play roles in resistance to the oxidative stress caused by ammonia, thereby beneficial to adapt to a high ammonia condition (Wu et al., 2021). In our study, only the *M*. *harundinacea* and *Methanosarcina* highly expressed energy-dependent anti-oxidative stress genes (*sor* and *rbr*), which made them predominant methanogens at the N6 stage. The dominant *Methanothrix* and *Methanosarcina* yielded methane possibly through both acetate cleavage and CO_2_ reduction, and the CO_2_ reduction pathway might provide energy for anti-oxidative stress metabolism, thereby achieving ammonia-inhibition resistance. However, as the expression of genes in a pathway does not mean the pathway has metabolic activity, further studies are needed in the future to obtain direct evidence that the CO_2_ reduction pathway of the two dominant methanogens has activity. In addition, further studies using pure cultures of these methanogens should be conducted to obtain physiological and biochemical evidence of their ammonia resistance.

As for the bacteria, *Petrimonas*, *Proteiniphilum*, unclassified Lentimicrobiaceae, *Syntrophomonas*, and *Sphaerochaeta* showed a significantly positive correlation trend with TAN concentration (Figure 2). As discussed above, methanogens need electrons from syntrophic acetate-oxidizing bacteria for CO_2_ reduction. According to the metagenomic analysis, we found several bins with acetate-oxidizing potential in Firmicutes, such as unclassified Thermoanaerobacteraceae (Bin68), unclassified Clostridiales (Bin54), and *Tepidanaerobacter* (Bin10), etc., consistent with previous studies (Hattori, 2008; Westerholm et al., 2018). Interestingly, *Proteiniphilum*, the only dominant bacterial genus belonging to Bacteroidetes, also fully expressed the acetate oxidation pathway (Figure 5C). At present, it is believed that the function of *Proteiniphilum* species is only hydrolysis and fermentation, its acetate oxidation ability is a new discovery that has not been found in previous studies (Hahnke et al., 2016; Wu et al., 2021). It is reasonable to assume that *Proteiniphilum* represents a broad range of metabolic capabilities including hydrolysis and fermentation and acetate oxidation to gain more energy, so it can dominate in inhibitory environments. We found a complete glycine cleavage pathway in the genomes of isolated *P. acetatigenes* and *P. saccharofermentans* strains (data not shown). Isolates of *P. acetatigenes* were found able to reduce CO_2_ with H_2_ to produce acetate (Kim et al., 2020), suggesting the expression of the glycine cleavage pathway. Some syntrophic acetate oxidizers including *Thermacetogenium phaeum*, *Clostridium ultunense*, etc., are also able to act as reductive acetogens (Hattori, 2008). Therefore, based on our analysis results and these reports, *Proteiniphilum* in our acetate-fed chemostat largely acted as syntrophic acetate oxidizers. Moreover, *Proteiniphilum* highly expressed the genes for energy-free anti-oxidative stress, making it more adaptable to ammonia stress, consistent with previous studies (Wu et al., 2021). Multiple studies have shown that *Proteiniphilum* can prevail in inhibitory conditions, such as high ammonia, high salt, and high oxygen (He et al., 2017; Lee et al., 2019; Wu et al., 2021). To further determine the acetate oxidation capacity of *Proteiniphilum*, further studies using pure monoculture of *Proteiniphilum* or co-culture with methanogens can be performed in the future.

## 5. Conclusion

This study revealed the microbial community composition and methanogenesis metabolic networks in the acetate-fed chemostat under high TAN conditions. Through gradual acclimation, the chemostat was able to operate stably at a TAN concentration of 6 g L^−1^. *Methanothrix* predominated in methanogens all the time, in which the dominant species was gradually replaced from *M. soehngenii* to *M. harundinacea* with the increasing TAN, suggesting a stronger TAN tolerance of *M. harundinacea*. Furthermore, we found that the genes involved in the CO_2_-reducing methanogenesis pathway in *M. harundinacea* had high expression levels, suggesting the possibility that *Methanothrix* might directly use extracellular electrons from syntrophic bacteria for reducing CO_2_ to CH_4_. During acclimation, the bacterial community underwent significant species turnover. We found diverse potential acetate-oxidizing bacteria under high TAN conditions, including the only dominant bacteria, *Proteiniphilum*, which highly expressed genes for acetate degradation. Thus, *Proteiniphilum* populations in the chemostat were possibly novel acetate degraders. The findings of this study provide a basis for improving ammonia tolerance and operational stability of AD.

## Data availability statement

The datasets presented in this study can be found in online repositories. The names of the repository/repositories and accession number(s) can be found at: https://www.ncbi.nlm.nih.gov/, PRJNA524473; http://bigd.big.ac.cn/gsa, CRA008529.

## Author contributions

GF operated reactor, analyzed data, and wrote the manuscript. YZ and H-ZW operated reactor and analyzed data. Y-TC analyzed the data. Y-QT directed experiments, data analysis, and manuscript revision. All authors contributed to the article and approved the submitted version.

## Funding

This study was financially supported by the National Key R&D Program of China (2022YFE0108500) and the National Natural Science Foundation of China (No. 51678378).

## Conflict of interest

The authors declare that the research was conducted in the absence of any commercial or financial relationships that could be construed as a potential conflict of interest.

## Publisher’s note

All claims expressed in this article are solely those of the authors and do not necessarily represent those of their affiliated organizations, or those of the publisher, the editors and the reviewers. Any product that may be evaluated in this article, or claim that may be made by its manufacturer, is not guaranteed or endorsed by the publisher.
