# Supplementary material for: Proteiniphilum and Methanothrix harundinacea became dominant acetate utilizers in a methanogenic reactor operated under strong ammonia stress
Source: Front Microbiol. 2023 Jan 6;13:1098814. doi: 10.3389/fmicb.2022.1098814 (PMC9853277; doi:10.3389/fmicb.2022.1098814)

Fig. S1 Structure diagram of completely stirred tank reactor (CSTR).

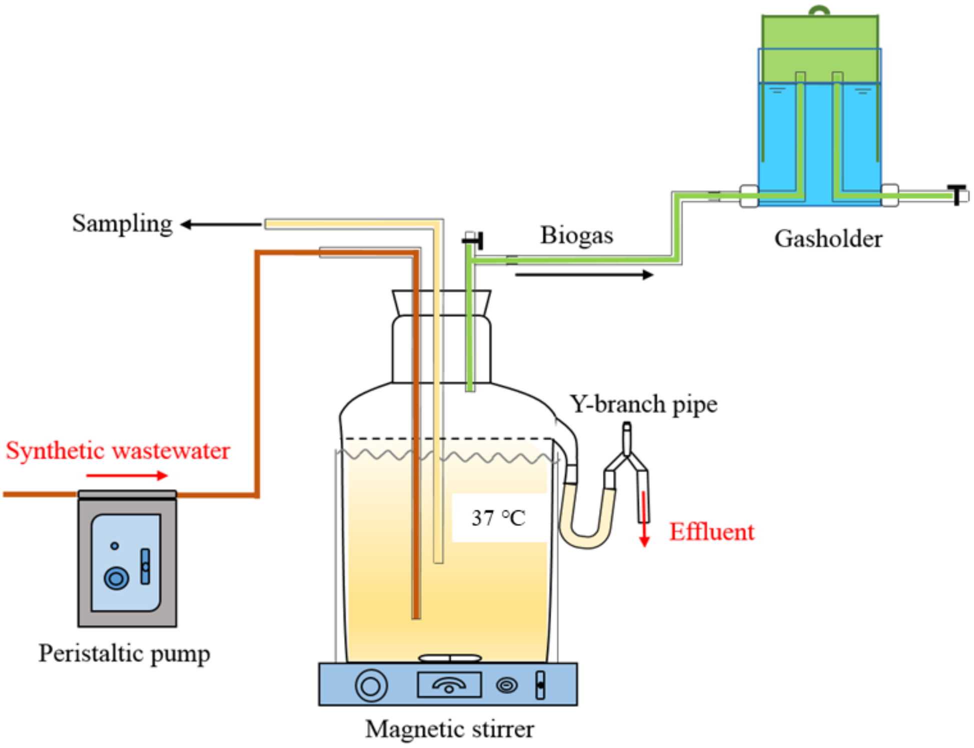

Fig. S2 Light (A) and fluorescent (B) field of microscopic views of the microbial communities in acetate-fed anaerobic chemostat operated under different TAN concentrations.

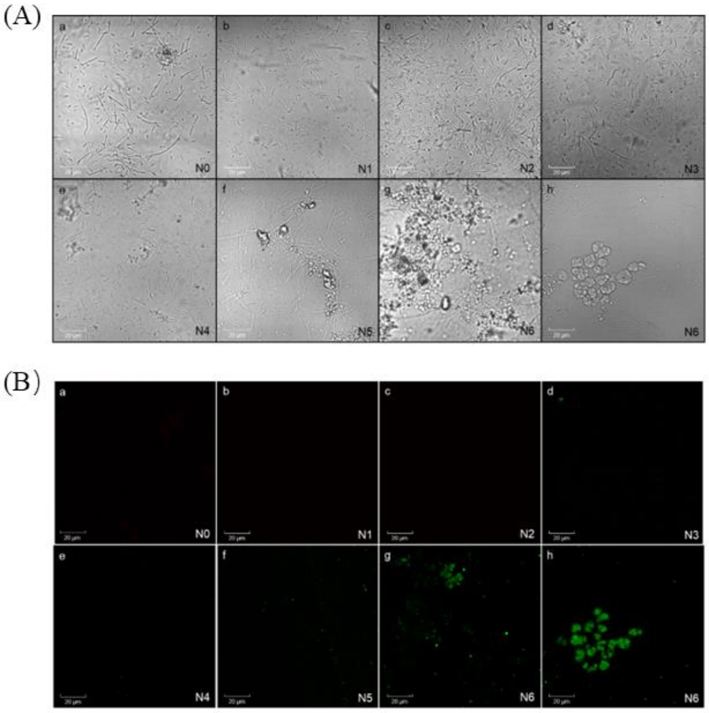

Fig. S3 The PCA analysis based on OTUs abundance.

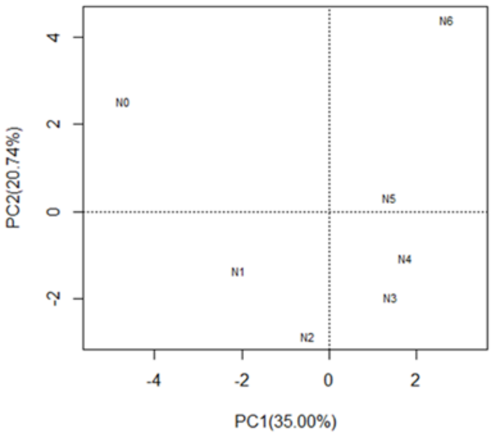

Fig. S4 Relative abundance of bacteria and archaea under different TAN concentrations.

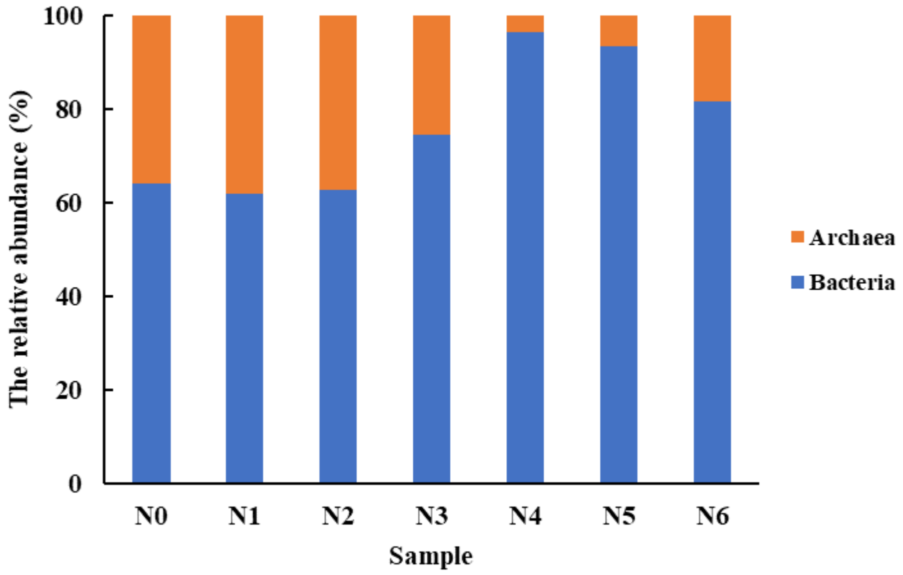

Fig. S5 Phylogenetic analyses of archaeal OTUs at the species level in the acetate-fed and ammonia-inhibited reactor based on 16S rRNA gene amplicon sequencing.

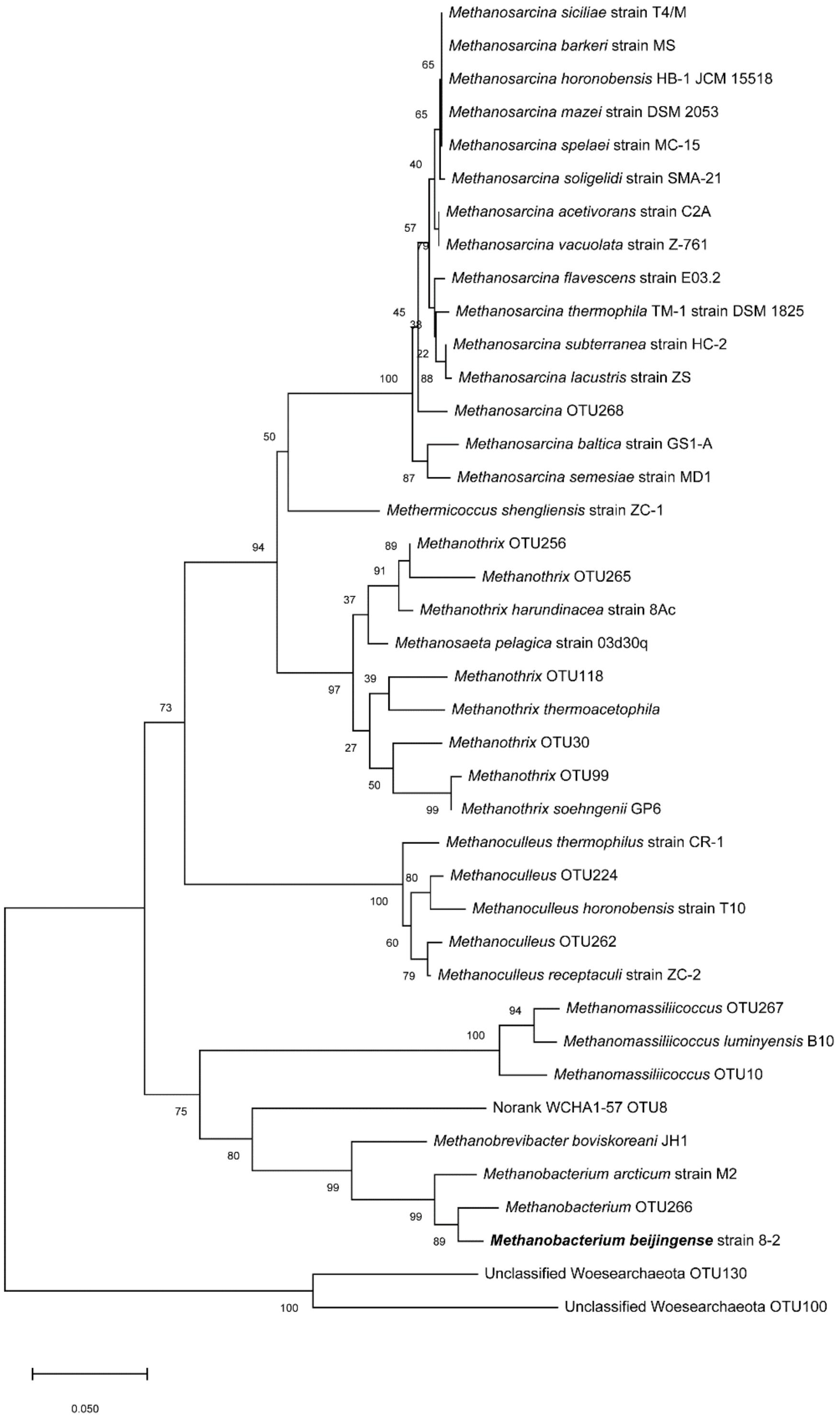

Fig. S6 Relative abundance of bacterial genera (A) and archaeal species (B) based on 16S rRNA gene amplicon sequencing in the acetate-fed chemostat under different TAN concentrations.

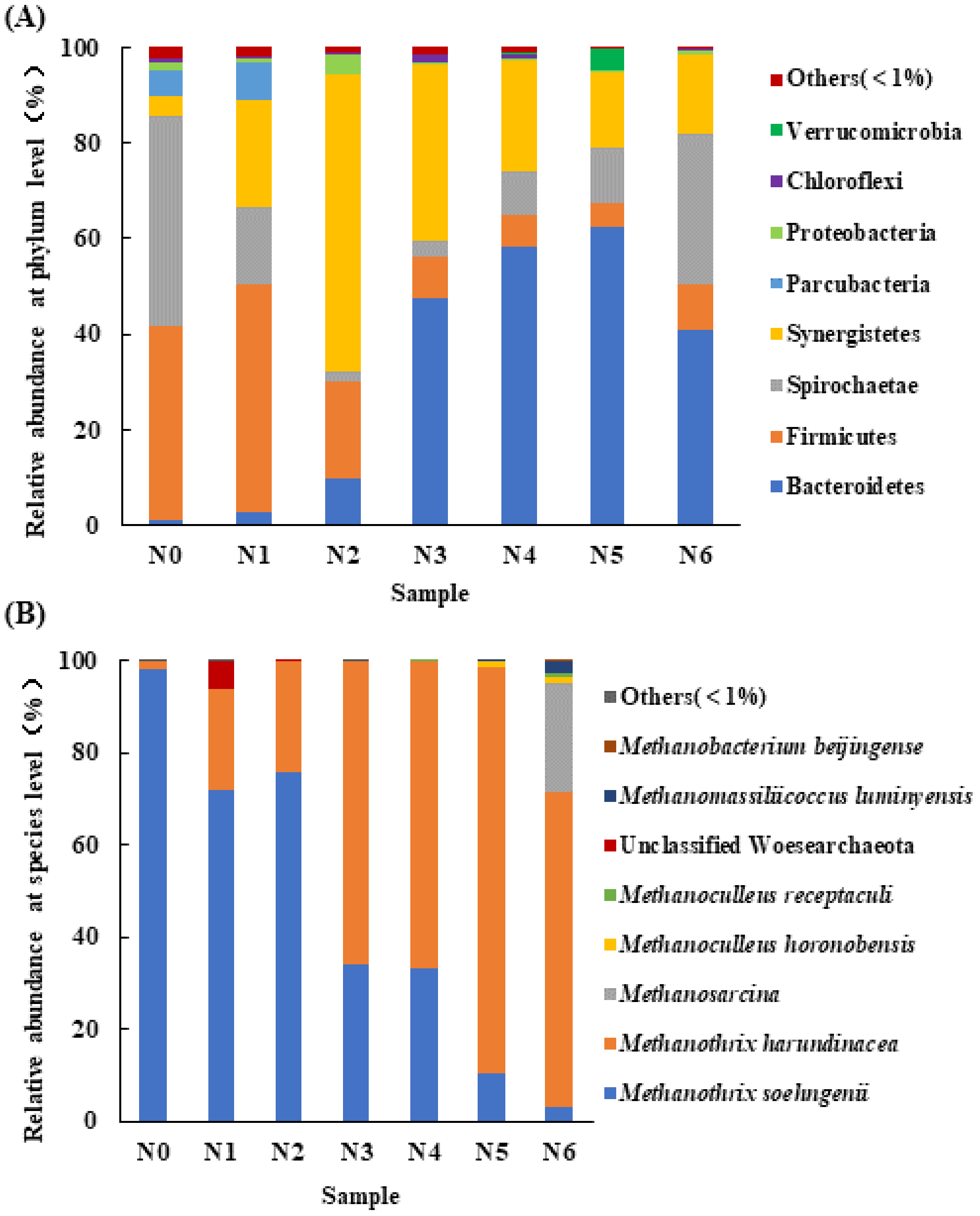

Fig. S7 Phylogenetic analyses of dominant genomic bins of bacteria in the acetate-fed chemostat at the N6 stage.

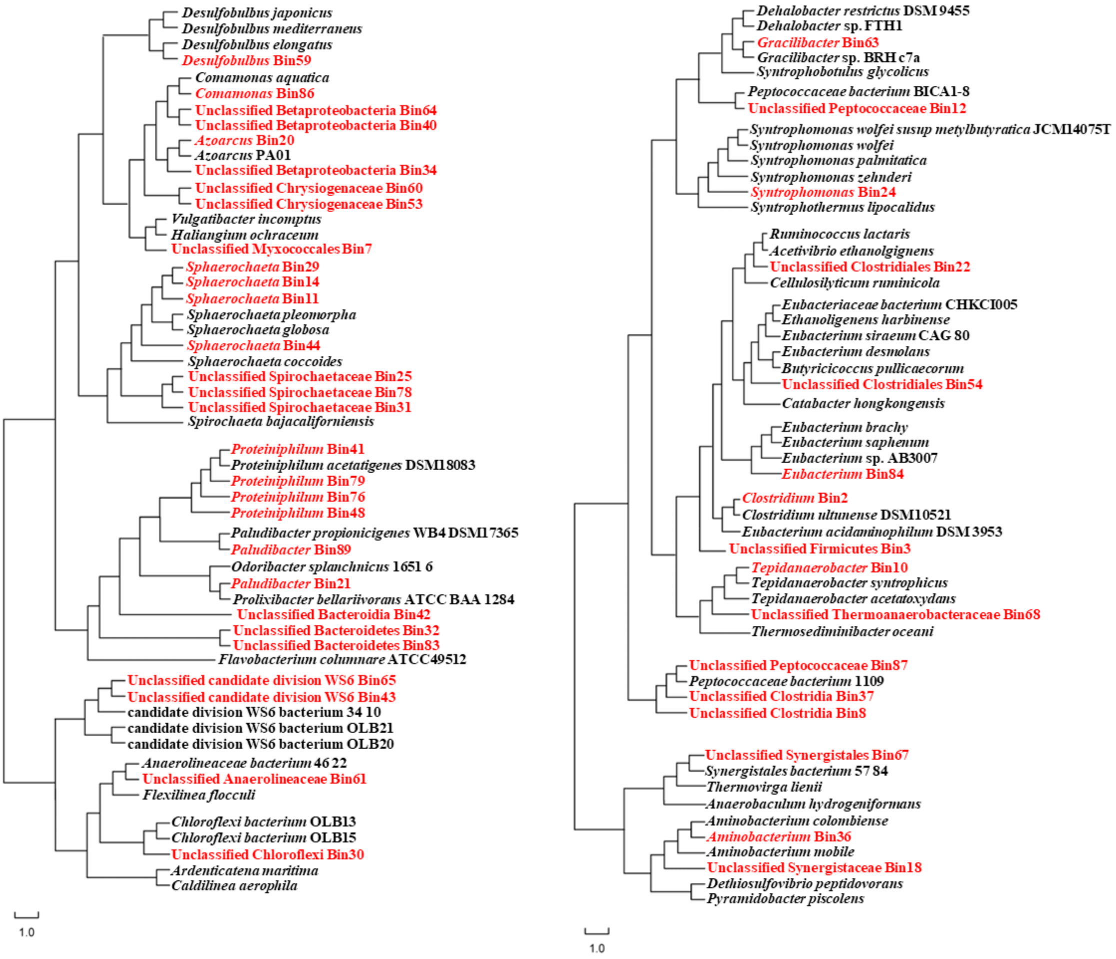

Fig. S8 Phylogenetic analyses of dominant genomic bins of archaea in the acetate-degrading chemostat at the N6 stage.

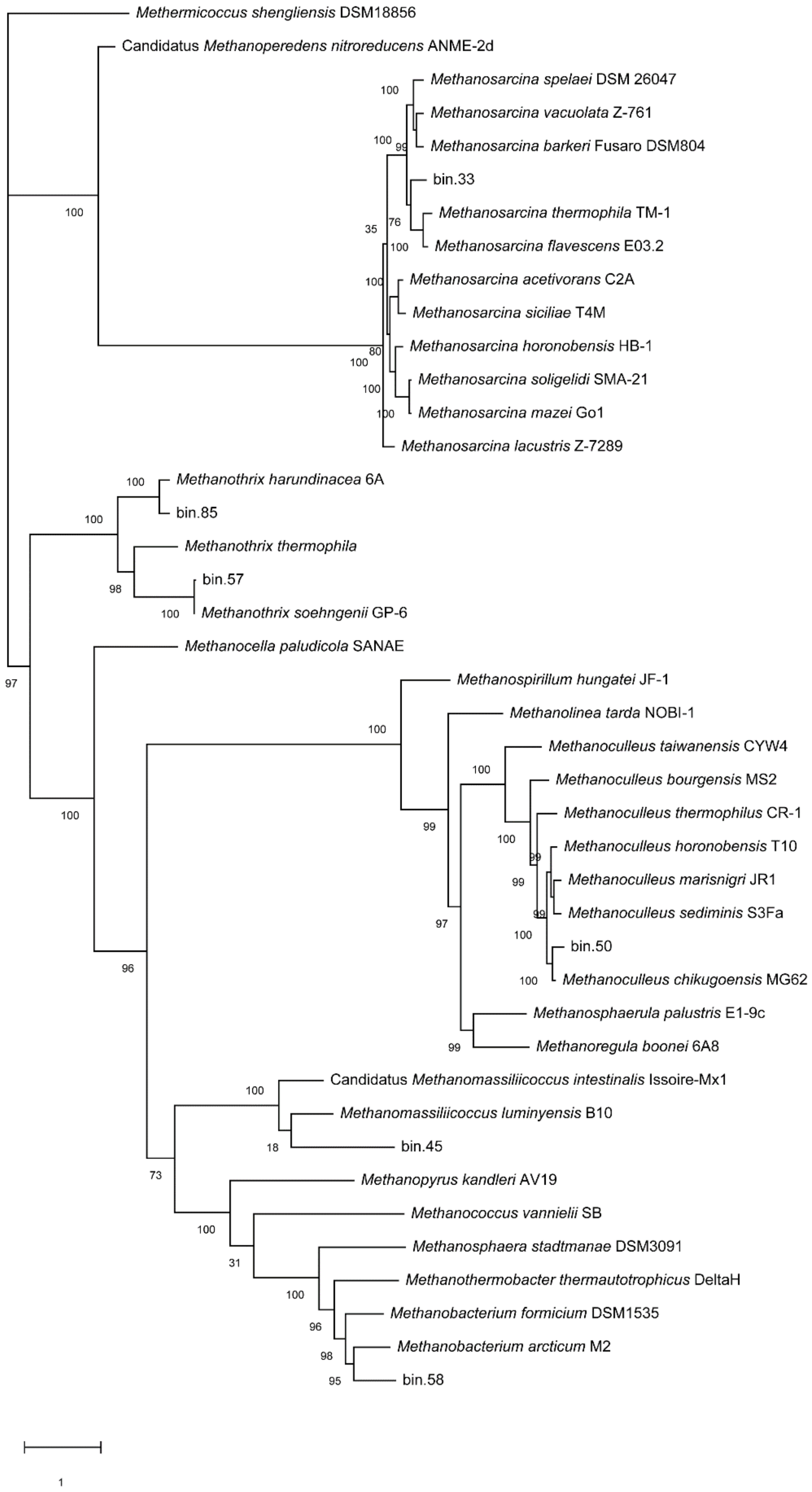

Fig. S9 The relative abundance of bacteria and archaea in the N6 stage. MG: estimated from their metagenomic coverage calculated as the percentage of metagenomics reads mapped to bacterial or archaeal bins relative to the total reads mapped to all constructed bacterial and archaeal bins. MT: estimated as the percentage of metatranscriptomic reads mapped to bacterial or archaeal bins relative to the total reads mapped to all constructed bacterial and archaeal bins. MT1, MT reads of sampling time point 1; MT2, MT reads of sampling time point 2.

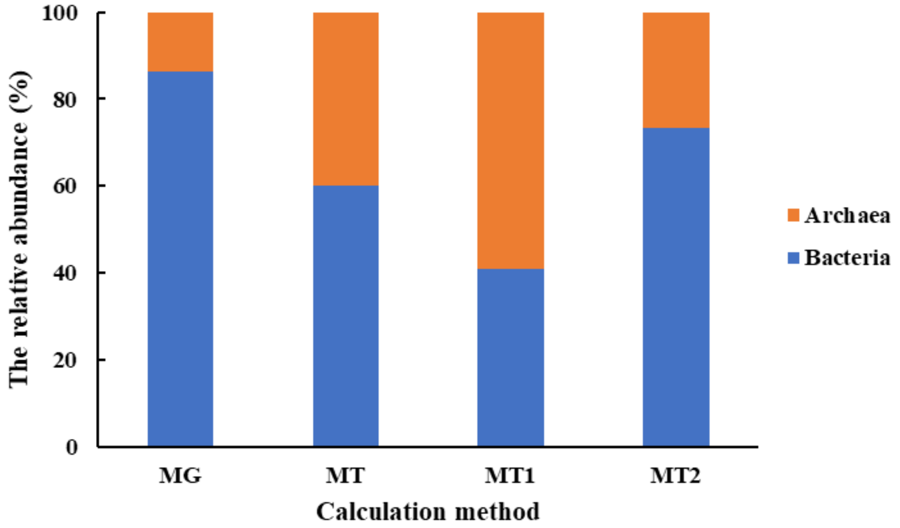

Fig. S10 Blast of key functional genes involved in acetate oxidation of 45 bins in the acetate-fed chemostat at the N6 stage.

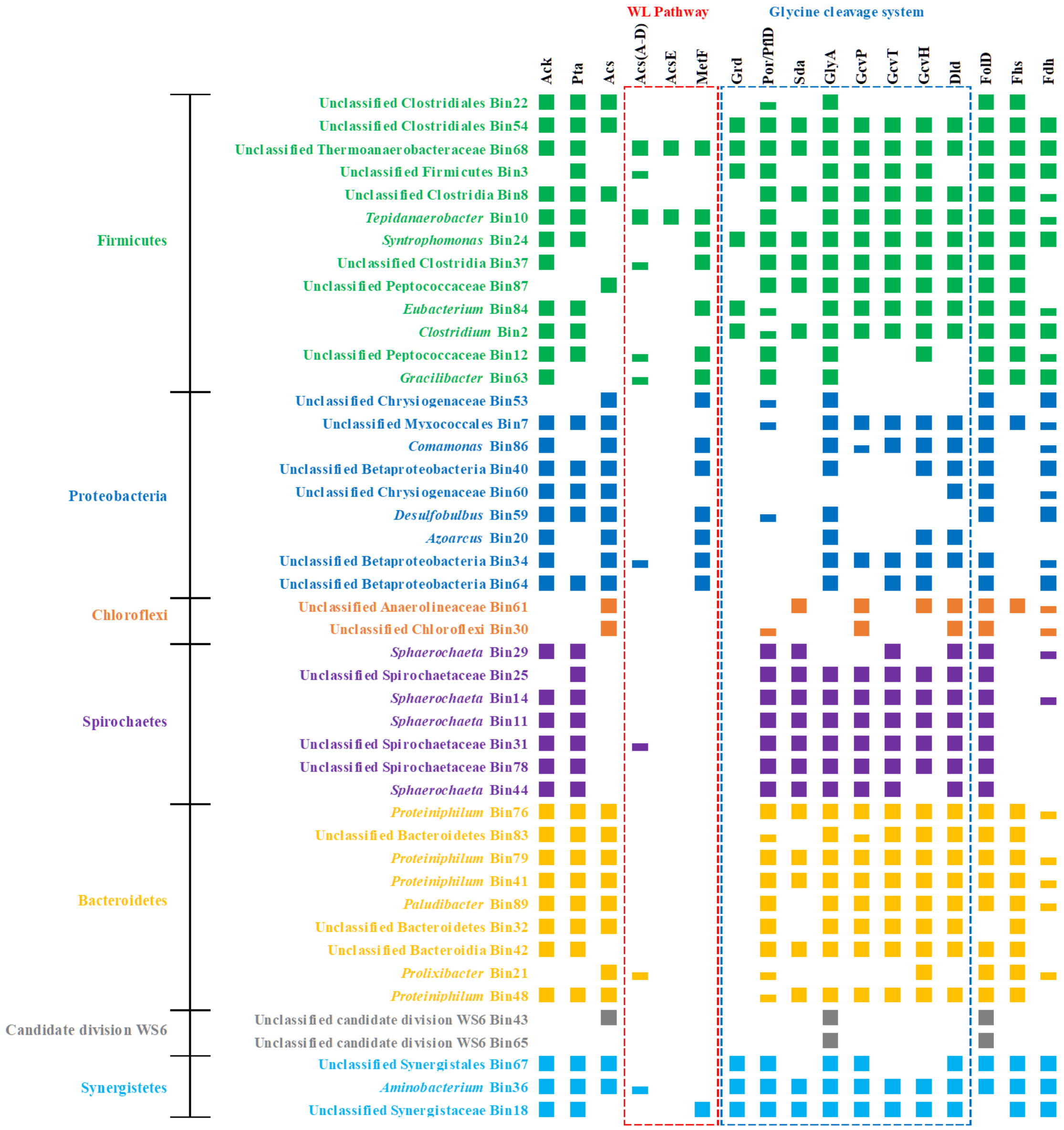

Fig. S11 Blast of key functional genes involved in H_2/formate metabolism and electron transfer of 45 bins in the acetate-fed chemostat at the N6 stage.

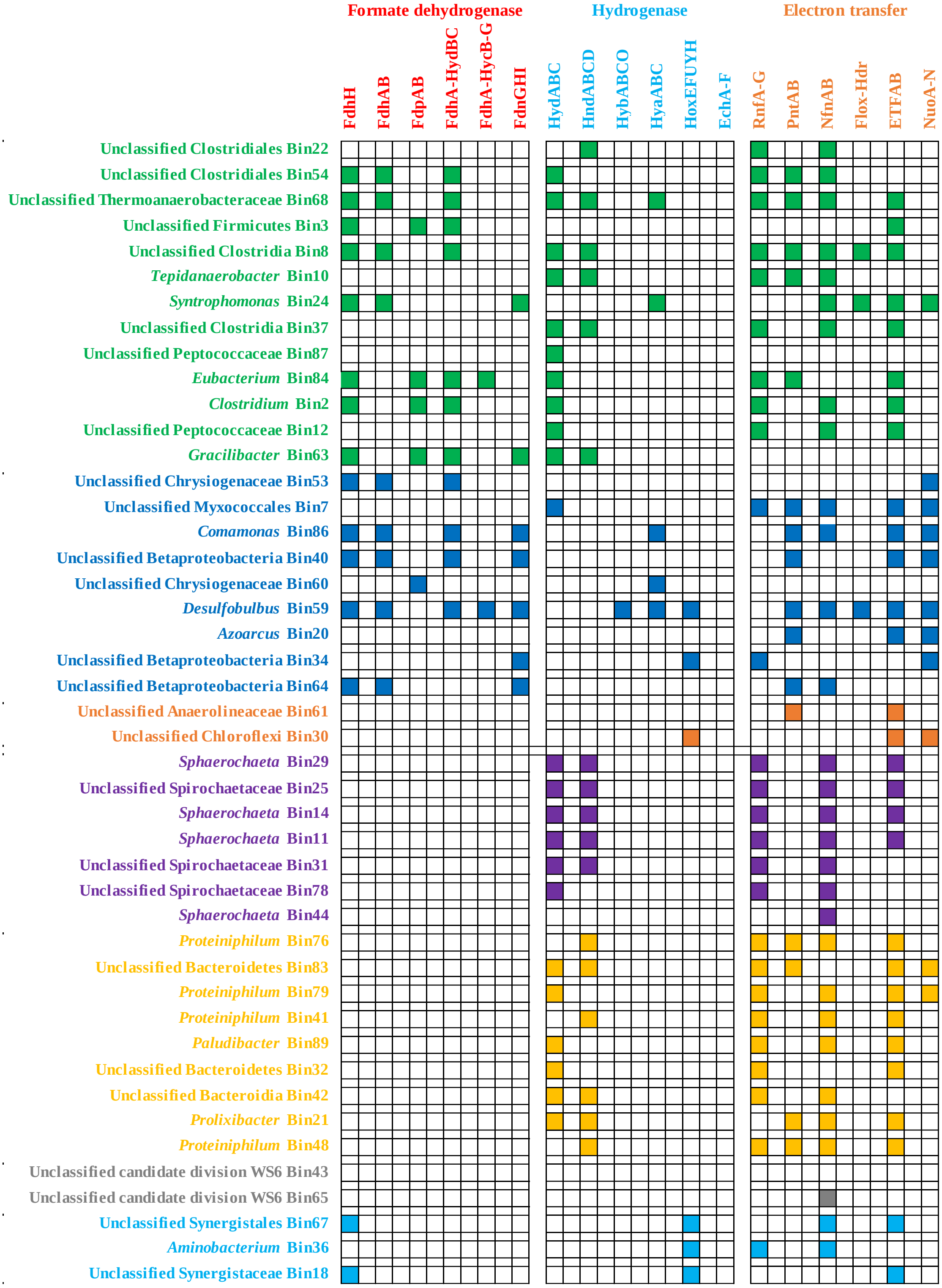

Supplement: Supplementary file 2 [file Image_1.PDF]
