# Supplementary material for: Proteiniphilum and Methanothrix harundinacea became dominant acetate utilizers in a methanogenic reactor operated under strong ammonia stress
Source: Front Microbiol. 2023 Jan 6;13:1098814. doi: 10.3389/fmicb.2022.1098814 (PMC9853277; doi:10.3389/fmicb.2022.1098814)
Supplement: Supplementary file 3 [file Table_1.DOCX]

Table S1 General characteristics of genomic bins of bacteria and methanogens in this study

| Bin ID | Phylum | Genus | Completeness (%) | Contamination (%) | Coverage |
| --- | --- | --- | --- | --- | --- |
|  |  |  |  |  |  |
| Bin59 | Proteobacteria | *Desulfobulbus* | 100 | 0 | 7.76 |
| Bin86 |  | *Comamonas* | 97.51 | 0.5 | 11.11 |
| Bin64 |  | Unclassified Betaproteobacteria | 75.94 | 1.82 | 3.14 |
| Bin40 |  | Unclassified Betaproteobacteria | 98.46 | 0.07 | 8.95 |
| Bin20 |  | *Azoarcus* | 88.71 | 2.05 | 7.43 |
| Bin34 |  | Unclassified Betaproteobacteria | 93.52 | 0.49 | 4.57 |
| Bin60 |  | Unclassified Chrysiogenaceae | 91.87 | 0.81 | 14.07 |
| Bin53 |  | Unclassified Chrysiogenaceae | 98.37 | 1.02 | 266.75 |
| Bin7 |  | Unclassified Myxococcales | 78.36 | 0.65 | 18.5 |
| Bin29 | Spirochaetes | *Sphaerochaeta* | 95.33 | 0 | 178.58 |
| Bin14 |  | *Sphaerochaeta* | 91.36 | 3.41 | 14.85 |
| Bin11 |  | *Sphaerochaeta* | 96.48 | 1.15 | 16.74 |
| Bin44 |  | *Sphaerochaeta* | 82.46 | 2.37 | 3.17 |
| Bin25 |  | Unclassified Spirochaetesceae | 93.11 | 2.46 | 27.02 |
| Bin78 |  | Unclassified Spirochaetesceae | 100 | 1.14 | 4.62 |
| Bin31 |  | Unclassified Spirochaetesceae | 96.52 | 3.41 | 8.34 |
| Bin41 | Bacteroidetes | *Proteiniphilum* | 97.27 | 0.55 | 145.08 |
| Bin79 |  | *Proteiniphilum* | 99.73 | 0.55 | 151.19 |
| Bin76 |  | *Proteiniphilum* | 100 | 0 | 211.25 |
| Bin48 |  | *Proteiniphilum* | 95.68 | 1.82 | 6.17 |
| Bin89 |  | *Paludibacter* | 99.73 | 0.54 | 133.97 |
| Bin21 |  | *Prolixibacter* | 72.85 | 2.97 | 7.35 |
| Bin42 |  | Unclassified Bacteroidia | 95.14 | 1.43 | 69.16 |
| Bin32 |  | Unclassified Bacteroidetes | 80.11 | 1.61 | 109.59 |
| Bin83 |  | Unclassified Bacteroidetes | 97.31 | 1.88 | 184.99 |
| Bin65 | Candidate  division WS6 | Unclassified candidate division WS6 | 76.41 | 1.72 | 6.59 |
| Bin43 |  | Unclassified candidate division WS6 | 77.27 | 0 | 31.51 |
| Bin61 | Chloroflexi | Unclassified Anaerolineaceae | 90 | 6.46 | 9.26 |
| Bin30 |  | Unclassified Chloroflexi | 80.57 | 0.91 | 2.46 |
| Bin67 | Synergistetes | Unclassified Synergistales | 100 | 1.69 | 190.02 |
| Bin36 |  | *Aminobacterium* | 100 | 1.69 | 6.1 |
| Bin18 |  | Unclassified Synergistaceae | 77.47 | 1.69 | 2.97 |
| Bin63 | Firmicutes | *Gracilibacter* | 96.55 | 2.03 | 4.37 |
| Bin12 |  | Unclassified Peptococcaceae | 85.68 | 1.46 | 6.63 |
| Bin24 |  | *Syntrophomonas* | 85.59 | 0 | 9.61 |
| Bin22 |  | Unclassified Clostridiales | 99.33 | 0.56 | 129.12 |
| Bin54 |  | Unclassified Clostridiales | 88.65 | 0.71 | 60.74 |
| Bin84 |  | *Eubacterium* | 97.16 | 0 | 4.57 |
| Bin2 |  | *Clostridium* | 96.7 | 1.47 | 4.61 |
| Bin3 |  | Unclassified Firmicutes | 86.27 | 0 | 26.4 |
| Bin10 |  | *Tepidanaerobacter* | 92.31 | 0 | 15.13 |
| Bin68 |  | Unclassified Thermoanaerobacteraceae | 99.04 | 1.49 | 13.78 |
| Bin87 |  | Unclassified Peptococcaceae | 91.18 | 2.94 | 7.36 |
| Bin37 |  | Unclassified Clostridia | 95.59 | 2.29 | 6.94 |
| Bin8 |  | Unclassified Clostridia | 90.2 | 1.96 | 11.56 |
| Bin58 | Euryarchaeota | *Methanobacterium* | 99.2 | 0 | 6.51 |
| Bin45 |  | *Methanomassiliicoccus* | 87.63 | 1.61 | 4.05 |
| Bin50 |  | *Methanoculleus* | 87.5 | 1.63 | 26.07 |
| Bin57 |  | *Methanothrix concilii* | 94.12 | 0.65 | 25.67 |
| Bin85 |  | *Methanothrix harundinacea* | 100 | 0 | 286.63 |
| Bin33 |  | *Methanosarcina* | 98.86 | 0.98 | 50.9 |

Table S2 Key genes involved in methanogenetic pathways

| Function | Abbreviation |
| --- | --- |
| Hydrogenotrophic methanogenetic pathways | |
| Formylmethanofuran dehydrogenase, subunit A | Fmd |
| Formylmethanofuran dehydrogenase, subunit B |  |
| Formylmethanofuran dehydrogenase, subunit C |  |
| Formylmethanofuran dehydrogenase, subunit D |  |
| Formylmethanofuran dehydrogenase, subunit E |  |
| Formylmethanofuran dehydrogenase, subunit F |  |
| Formylmethanofuran dehydrogenase, subunit G |  |
| Formylmethanofuran-tetrahydromethanopterin N-formyltransferase | Ftr |
| Methenyltetrahydromethanopterin cyclohydrolase | Mch |
| Methylenetetrahydromethanopterin dehydrogenase | Mtd |
| Coenzyme F_420_-dependent N5, N10-methenyltetrahydromethanopterin reductase | Mer |
| Tetrahydromethanopterin S-methyltransferase, subunit A | Mtr |
| Tetrahydromethanopterin S-methyltransferase, subunit B |  |
| Tetrahydromethanopterin S-methyltransferase, subunit C |  |
| Tetrahydromethanopterin S-methyltransferase, subunit D |  |
| Tetrahydromethanopterin S-methyltransferase, subunit E |  |
| Tetrahydromethanopterin S-methyltransferase, subunit F |  |
| Tetrahydromethanopterin S-methyltransferase, subunit G |  |
| Tetrahydromethanopterin S-methyltransferase, subunit H |  |
| Methyl-CoM reductase alpha subunit | Mcr |
| Methyl-CoM reductase beta subunit |  |
| Methyl-CoM reductase gamma subunit |  |
| Methyl-coenzyme M reductase operon protein D |  |
| Methyl-coenzyme M reductase operon protein C |  |
| Acetotrophic methanogenetic pathways | |
| Acetate kinase | Ack |
| Phosphate acetyltransferase | Pta |
| Acetyl-CoA synthetase | Acs |
| Acetyl-CoA decarbonylase/synthase complex, subunit alpha | Cdh |
| Acetyl-CoA decarbonylase/synthase complex, subunit beta |  |
| Acetyl-CoA decarbonylase/synthase complex, subunit gamma |  |
| Acetyl-CoA decarbonylase/synthase complex, subunit delta |  |
| Acetyl-CoA decarbonylase/synthase complex, subunit epsilon |  |
| Tetrahydromethanopterin S-methyltransferase, subunit A | Mtr |
| Tetrahydromethanopterin S-methyltransferase, subunit B |  |
| Tetrahydromethanopterin S-methyltransferase, subunit C |  |
| Tetrahydromethanopterin S-methyltransferase, subunit D |  |
| Tetrahydromethanopterin S-methyltransferase, subunit E |  |
| Tetrahydromethanopterin S-methyltransferase, subunit F |  |

| Tetrahydromethanopterin S-methyltransferase, subunit G | Mtr |
| --- | --- |
| Tetrahydromethanopterin S-methyltransferase, subunit H |  |
| Methyl-CoM reductase alpha subunit | Mcr |
| Methyl-CoM reductase beta subunit |  |
| Methyl-CoM reductase gamma subunit |  |
| Methyl-coenzyme M reductase operon protein D |  |
| Methyl-coenzyme M reductase operon protein C |  |
| Methylotrophic methanogenetic pathways | |
| Methylcobamide: CoM methyltransferase isozyme M | Mta |
| Methyl-CoM reductase alpha subunit | Mcr |
| Methyl-CoM reductase beta subunit |  |
| Methyl-CoM reductase gamma subunit |  |
| Methyl-coenzyme M reductase operon protein D |  |
| Methyl-coenzyme M reductase operon protein C |  |

Table S3 Genes involved in energy conservation found in methanogens

| Function | Abbreviation |
| --- | --- |
| Energy-converting [NiFe] hydrogenase subunit A | Ech A-F |
| Energy-converting [NiFe] hydrogenase subunit B |  |
| Energy-converting [NiFe] hydrogenase subunit C |  |
| Energy-converting [NiFe] hydrogenase subunit D |  |
| Energy-converting [NiFe] hydrogenase subunit E |  |
| Energy-converting [NiFe] hydrogenase subunit F |  |
| Energy-converting hydrogenase A, Eha complex subunit A | EhaA-T |
| Energy-converting hydrogenase A, Eha complex subunit B |  |
| Energy-converting hydrogenase A, Eha complex subunit C |  |
| Energy-converting hydrogenase A, Eha complex subunit D |  |
| Energy-converting hydrogenase A, Eha complex subunit E |  |
| Energy-converting hydrogenase A, Eha complex subunit F |  |
| Energy-converting hydrogenase A, Eha complex subunit G |  |
| Energy-converting hydrogenase A, Eha complex subunit H |  |
| Energy-converting hydrogenase A, Eha complex subunit I |  |
| Energy-converting hydrogenase A, Eha complex subunit J |  |
| Energy-converting hydrogenase A, Eha complex subunit K |  |
| Energy-converting hydrogenase A, Eha complex subunit L |  |
| Energy-converting hydrogenase A, Eha complex subunit M |  |
| Energy-converting hydrogenase A, Eha complex subunit N |  |
| Energy-converting hydrogenase A, Eha complex subunit O |  |
| Energy-converting hydrogenase A, Eha complex subunit P |  |
| Energy-converting hydrogenase A, Eha complex subunit Q |  |
| Energy-converting hydrogenase A, Eha complex subunit R |  |
| Energy-converting hydrogenase A, Eha complex subunit S |  |
| Energy-converting hydrogenase A, Eha complex subunit T |  |
| Energy-converting hydrogenase B, Eha complex subunit A | EhbA-Q |
| Energy-converting hydrogenase B, Eha complex subunit B |  |
| Energy-converting hydrogenase B, Eha complex subunit C |  |
| Energy-converting hydrogenase B, Eha complex subunit D |  |
| Energy-converting hydrogenase B, Eha complex subunit E |  |
| Energy-converting hydrogenase B, Eha complex subunit F |  |
| Energy-converting hydrogenase B, Eha complex subunit G |  |
| Energy-converting hydrogenase B, Eha complex subunit H |  |
| Energy-converting hydrogenase B, Eha complex subunit I |  |
| Energy-converting hydrogenase B, Eha complex subunit J |  |
| Energy-converting hydrogenase B, Eha complex subunit K |  |
| Energy-converting hydrogenase B, Eha complex subunit L |  |
| Energy-converting hydrogenase B, Eha complex subunit M |  |
| Energy-converting hydrogenase B, Eha complex subunit N |  |
| Energy-converting hydrogenase B, Eha complex subunit O |  |
| Energy-converting hydrogenase B, Eha complex subunit P |  |
| Energy-converting hydrogenase B, Eha complex subunit Q |  |
| F_420_-non-reducing [NiFe] hydrogenase subunit A | MvhAGD |
| F_420_-non-reducing [NiFe] hydrogenase subunit G |  |
| F_420_-non-reducing [NiFe] hydrogenase subunit D |  |
| Methano-phenazine-reducing [NiFe] hydrogenase subunit A | VhoAGC |
| Methano-phenazine-reducing [NiFe] hydrogenase subunit C |  |
| Methano-phenazine-reducing [NiFe] hydrogenase subunit G |  |
| Heterodisulfide reductase iron-sulfur subunit A | HdrABC |
| Heterodisulfide reductase subunit B |  |
| Heterodisulfide reductase iron-sulfur subunit C |  |
| Heterodisulfide reductase iron-sulfur subunit A2 | HdrA2B2C2 |
| Heterodisulfide reductase subunit B2 |  |
| Heterodisulfide reductase iron-sulfur subunit C2 |  |
| Methanophenazine-dependent heterodisulphide reductase subunit D | HdrDE |
| Methanophenazine-dependent heterodisulphide reductase subunit E |  |
| F_420_-reducing hydrogenase subunit A | FrhABG |
| F_420_-reducing hydrogenase subunit B |  |
| F_420_-reducing hydrogenase subunit D |  |
| F_420_-reducing hydrogenase subunit G |  |
| Energy-conserving F_420_H_2_-dehydrogenase complex subunit A | FpoA-O |
| Energy-conserving F_420_H_2_-dehydrogenase complex subunit B |  |
| Energy-conserving F_420_H_2_-dehydrogenase complex subunit C |  |
| Energy-conserving F_420_H_2_-dehydrogenase complex subunit D |  |
| Energy-conserving F_420_H_2_-dehydrogenase complex subunit H |  |
| Energy-conserving F_420_H_2_-dehydrogenase complex subunit I |  |
| Energy-conserving F_420_H_2_-dehydrogenase complex subunit J |  |
| Energy-conserving F_420_H_2_-dehydrogenase complex subunit K |  |
| Energy-conserving F_420_H_2_-dehydrogenase complex subunit L |  |
| Energy-conserving F_420_H_2_-dehydrogenase complex subunit M |  |
| Energy-conserving F_420_H_2_-dehydrogenase complex subunit N |  |
| Energy-conserving F_420_H_2_-dehydrogenase complex subunit O |  |
| Energy-conserving F_420_H_2_-dehydrogenase complex subunit F |  |
| Formate dehydrogenase subunit alpha | FdhAB |
| Formate dehydrogenase subunit beta |  |
| Na^+^/H^+^ antiporter | Nha |
| ATP synthase subunit A | Aha |
| ATP synthesis subunit B |  |
| ATP synthesis subunit C |  |
| ATP synthesis subunit D |  |
| ATP synthesis subunit E |  |
| ATP synthesis subunit F |  |
| ATP synthesis subunit I |  |

Table S4 Key genes involved in the acetate oxidation pathways

| Function | Abbreviation |
| --- | --- |
| Wood-Ljungdahl pathway | |
| Acetate kinase | Ack |
| Phosphate acetyltransferase/phosphotransacetylase | Pta |
| acetyl-CoA synthetase (ADP-forming) | Acs |
| Acetyl-CoA synthetase (AMP-forming) | Acs |
| Carbon monoxide dehydrogenase | AcsA |
| Acetyl CoA synthase catalytic subunit | AcsB |
| Corrinoid/iron-sulfur protein large subunit | AcsC |
| Corrinoid/iron-sulfur protein small subunit | AcsD |
| Carbon monoxide dehydrogenase accessory protein | CooC |
| Carbon monoxide dehydrogenase nickel-insertion accessory protein | CooS |
| Methyltetrahydrofolate:corrinoid/iron-sulfur protein methyltransferase | AcsE |
| Methylenetetrahydrofolate reductase | MetF |
| Methylenetetrahydrofolate dehydrogenase | Fold |
| Methenyltetrahydrofolate cyclohydrolase | Fold |
| Formate tetrahydrofolate ligase / formyltetrahydrofolate synthetase | Fhs |
| Formate dehydrogenase | Fdh |
| Glycine cleavage system | |
| Glycine/sarcosine/betaine reductase complex protein A | GrdA1 |
| Glycine/sarcosine/betaine reductase complex component A2 | GrdA2 |
| Glycine/sarcosine/betaine reductase complex component C subunit alpha | GrdC1 |
| Glycine/sarcosine/betaine reductase complex component C subunit beta | GrdC2 |
| Glycine reductase complex component B subunits alpha and beta | GrdB |
| Thiamine pyrophosphate protein domain protein TPP-binding | PorB |
| Pyruvate flavodoxin/ferredoxin oxidoreductase domain protein | PorA |
| Pyruvate ferredoxin/flavodoxin oxidoreductase, delta subunit | PorD |
| Pyruvate/ketoisovalerate oxidoreductase, gamma subunit | PorG |
| Pyruvate-ferredoxin/flavodoxin oxidoreductase | Por |
| Pyruvate-formate lyase | Pfld |
| Pyruvate-formate lyase activating enzyme |  |
| L-serine dehydratase, iron-sulfur-dependent, alpha subunit | SdaA |
| L-serine dehydratase, iron-sulfur-dependent, beta subunit | SdaB |
| Glycine hydroxymethyltransferase | GlyA |
| Glycine dehydrogenase subunit A | GcvPA |
| Glycine dehydrogenase subunit B | GcvPB |
| Glycine cleavage system T protein | GcvPT |
| Glycine cleavage system H protein | GcvPH |
| Dihydrolipoyl dehydrogenase | Dld |

Table S5 Genes involved in H_2_/formate metabolism, and electron transfer found in syntrophs

| Function | Abbreviation |
| --- | --- |
| Formate dehydrogenase | |
| Formate dehydrogenase H | FdhH |
| Formate dehydrogenase subunit alpha | FdhAB |
| Formate dehydrogenase subunit beta |  |
| Formate dehydrogenase subunit alpha | FdhA-HydBC |
| Electron-confurcating hydrogenase, nuoF-like subunit |  |
| Electron-confurcating hydrogenase, nuoE-like subunit |  |
| NADP-dependent formate dehydrogenase | FdpAB |
| Formate dehydrogenase subunit alpha | FdhA-hycB-G |
| Formate hydrogenlyase, ferredoxin subunit |  |
| Formate hydrogenlyase, antiporter subunit |  |
| Formate hydrogenlyase, membrane subunit |  |
| Formate hydrogenlyase, Ni,Fe hydrogenase large subunit |  |
| Formate hydrogenlyase, Fe-S subunit |  |
| Formate hydrogenlyase, Ni,Fe hydrogenase small subunit |  |
| Quinone-reducing formate dehydrogenase-N, catalytic subunit | FdnGHI |
| Quinone-reducing formate dehydrogenase N, Fe-S subunit |  |
| Quinone-reducing formate dehydrogenase-N, cytochrome b subunit |  |
| Hydrogenase | |
| (Fe) hydrogenase, large subunit HymC, putative | Hyd |
| Electron-confurcating hydrogenase, alpha subunit | HydABC |
| Electron-confurcating hydrogenase, beta subunit |  |
| Electron-confurcating hydrogenase, gamma subunit |  |
| NADP-reducing hydrogenase subunit HndA | HndABCD |
| NADP-reducing hydrogenase subunit HndB |  |
| NADP-reducing hydrogenase subunit HndC |  |
| NADP-reducing hydrogenase subunit HndD |  |
| quinol Fe-S-cluster-containing hydrogenase components 1 | HybABCO |
| quinol Polysulphide reductase |  |
| quinol Ni,Fe-hydrogenase I large subunit |  |
| quinol Ni,Fe-hydrogenase I small subunit |  |
| (Membrane-bound hydrogenase 1 large subunit) (NiFe hydrogenase) | HyaABC |
| (Membrane-bound hydrogenase 1 small subunit) (NiFe hydrogenase) |  |
| Ni/Fe-hydrogenase B-type cytochrome subunit |  |
| NAD-dependent hydrogenase (pentameric), nuoE-like subunit | HoxEFUYH |
| NAD-dependent hydrogenase (pentameric), nuoF-like subunit |  |
| NAD-dependent hydrogenase (pentameric), Fe-S-binding subunit |  |
| NAD-dependent hydrogenase (pentameric), frhG-like subunit |  |
| NAD-dependent hydrogenase (pentameric), catalytic subunit |  |
| Hydrogenase subunit A | EchA-F |
| Hydrogenase subunit B |  |
| Hydrogenase subunit C |  |
| Hydrogenase subunit D |  |
| Hydrogenase subunit E |  |
| Hydrogenase subunit F |  |
| Electron transfer | |
| Rnf electron transport complex subunit A | RnfA-G |
| Rnf electron transport complex subunit B |  |
| Rnf electron transport complex subunit C |  |
| Rnf electron transport complex subunit C2 |  |
| Rnf electron transport complex subunit D |  |
| Rnf electron transport complex subunit E |  |
| Rnf electron transport complex subunit G |  |
| NAD(P) transhydrogenase subunit beta | PntAB |
| NAD(P) transhydrogenase subunit alpha, part 2 |  |
| NAD(P) transhydrogenase subunit alpha, part 1 |  |
| NADH-quinone oxidoreductase subunit A | NuoA-N |
| NADH-quinone oxidoreductase subunit B |  |
| NADH-quinone oxidoreductase subunit C |  |
| NADH-quinone oxidoreductase subunit D |  |
| NADH-quinone oxidoreductase subunit E |  |
| NADH-quinone oxidoreductase subunit F |  |
| NADH-quinone oxidoreductase subunit G |  |
| NADH-quinone oxidoreductase subunit H |  |
| NADH-quinone oxidoreductase subunit I |  |
| NADH-quinone oxidoreductase subunit J |  |
| NADH-quinone oxidoreductase subunit K |  |
| NADH-quinone oxidoreductase subunit L |  |
| NADH-quinone oxidoreductase subunit N |  |
| ETF dehydrogenase, ETF beta subunit | EtfAB |
| ETF dehydrogenase, ETF alpha subunit |  |
| NADH-Fd:NADP^+^ oxidoreductase A | NfnAB |
| NADH-Fd:NADP^+^ oxidoreductase B |  |
| CoB--CoM heterodisulfide reductase subunit A | Flox-Hdr |
| CoB--CoM heterodisulfide reductase subunit B |  |
| CoB--CoM heterodisulfide reductase subunit C |  |
| CoB--CoM heterodisulfide reductase subunit A |  |
| CoB--CoM heterodisulfide reductase subunit B |  |
| CoB--CoM heterodisulfide reductase subunit C |  |
| Methyl-viologen-reducing hydrogenase, delta subunit |  |
| Coenzyme F420-non-reducing hydrogenase, subunit D |  |
| 4Fe-4S ferredoxin, iron-sulfur binding |  |
| 4Fe-4S ferredoxin, iron-sulfur binding |  |
| Oxidoreductase FAD/NAD(P)-binding |  |

Table S6 Key genes related to ROS-detoxification

|  | Abbrev | Function |
| --- | --- | --- |
| Energy free | Sod | superoxide oxide dismutase |
| Energy dependent | Sor | Superoxide reductase |
| Energy free | Cat | catalase |
|  | Pxd | peroxidase |
|  | Gpx | glutathione peroxidase |
|  | Prx | peroxiredoxins |
|  | Kat(G) | catalase-peroxidase |
| Energy dependent | Rbr | rubrerythrin |
